# Supplementary material for: Biomarkers of sickle cell nephropathy in Senegal
Source: PLoS One. 2022 Nov 21;17(11):e0273745. doi: 10.1371/journal.pone.0273745 (PMC9678278; doi:10.1371/journal.pone.0273745)
Supplement: S1 File — (PDF) [file pone.0273745.s001.pdf]

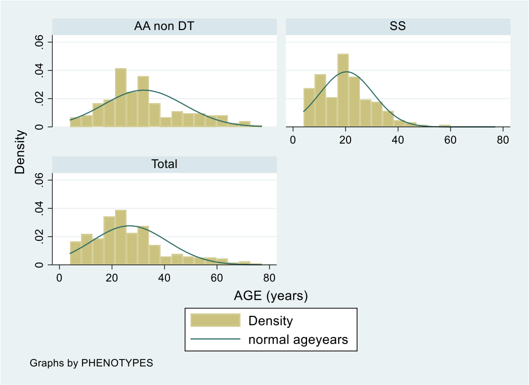

AA non DT
SS
Total
Density
AGE (years)
Density
normal ageyears
Graphs by PHENOTYPES

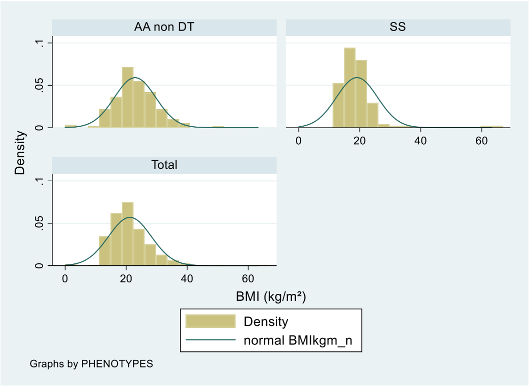

AA non DT
SS
Total
Density
BMI (kg/m²)
Density
normal BMIkgm_n
Graphs by PHENOTYPES

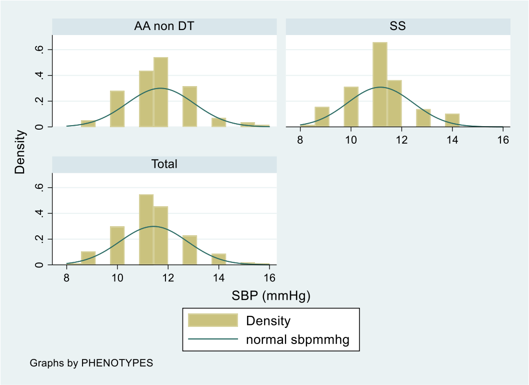
AA non DT
SS
Total
Density
SBP (mmHg)
Density
normal sbpmmhg
Graphs by PHENOTYPES

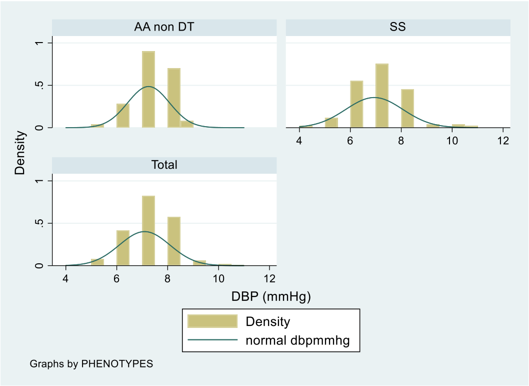
AA non DT
SS
Total
Density
DBP (mmHg)
Density
normal dbpmmhg
Graphs by PHENOTYPES

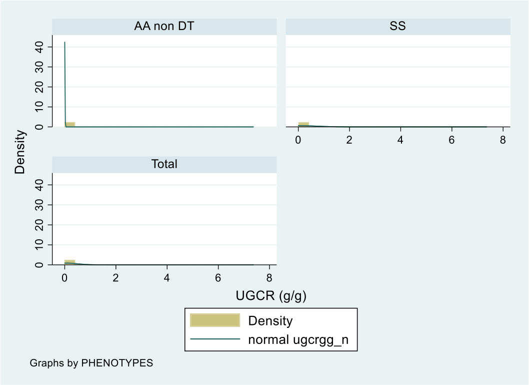
AA non DT
SS
Total
Density
UGCR (g/g)
Density
normal ugcrgg_n
Graphs by PHENOTYPES

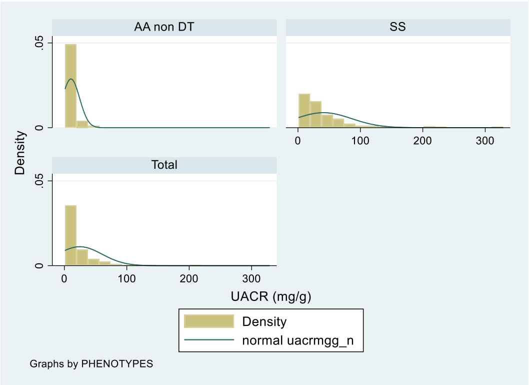
AA non DT
SS
Total
Density
UACR (mg/g)
Density
normal uacrmgg_n
Graphs by PHENOTYPES

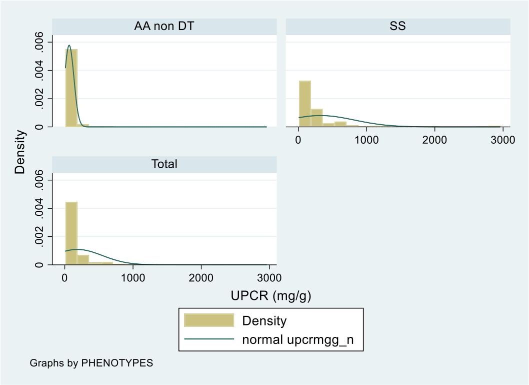
AA non DT
SS
Total
Density
0
.002
.004
.006
0
1000
2000
3000
UPCR (mg/g)
Density
normal upcrmgg_n
Graphs by PHENOTYPES

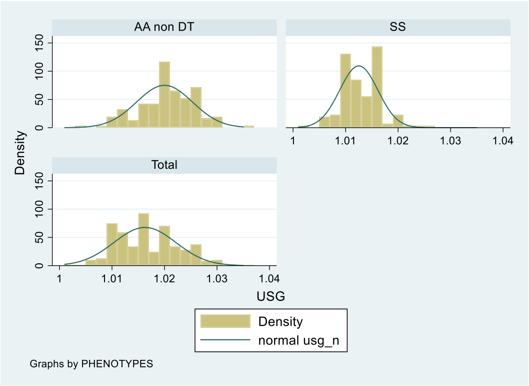
AA non DT
SS
Total
Density
0
50
100
150
1
1.01
1.02
1.03
1.04
USG
Density
normal usg_n
Graphs by PHENOTYPES

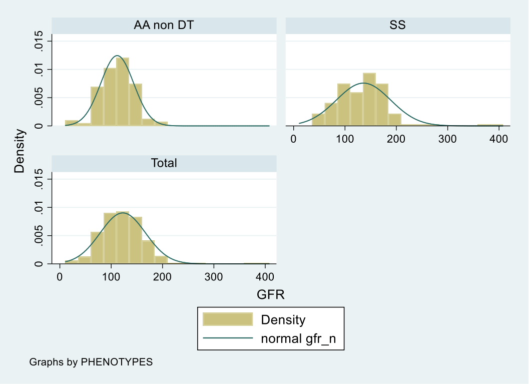
AA non DT
SS
Total
Density
0
.005
.01
.015
0
100
200
300
400
GFR
Density
normal gfr_n
Graphs by PHENOTYPES

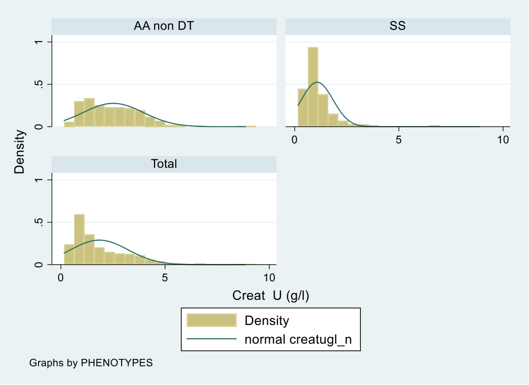
AA non DT
SS
Total
Density
0
.5
1
0
5
10
Creat U (g/l)
Density
normal creatugl_n
Graphs by PHENOTYPES

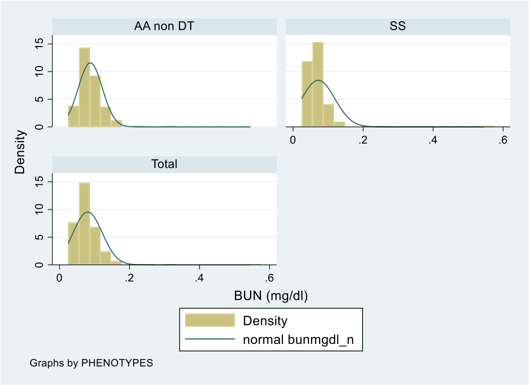
AA non DT
SS
Total
Density
BUN (mg/dl)
Density
normal bunmgdl_n
Graphs by PHENOTYPES

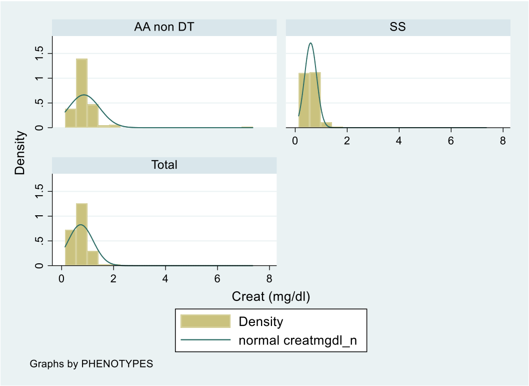
AA non DT
SS
Total
Density
Creat (mg/dl)
Density
normal creatmgdl_n
Graphs by PHENOTYPES

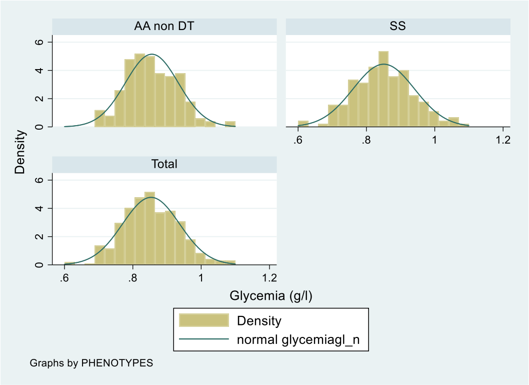
AA non DT
SS
Total
Density
Glycemia (g/l)
Density
normal glycemiagl_n
Graphs by PHENOTYPES

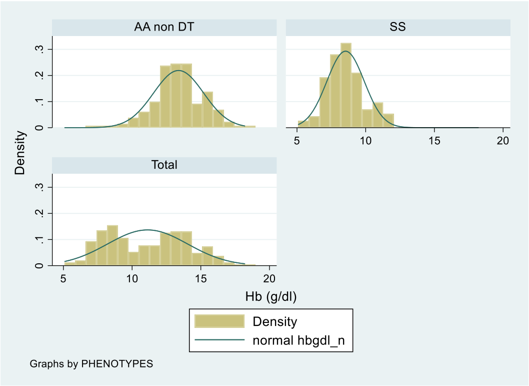
AA non DT
SS
Total
Density
Hb (g/dl)
Density
normal hbgdl_n
Graphs by PHENOTYPES

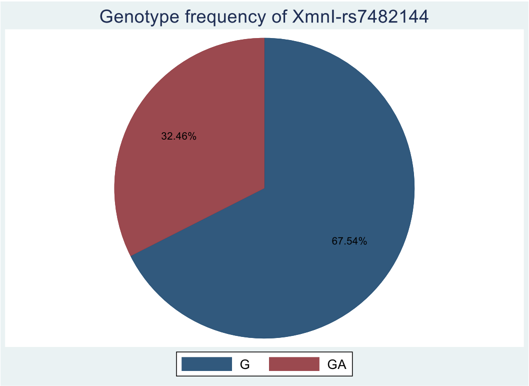
Genotype frequency of XmnI-rs7482144
32.46%
67.54%
G
GA

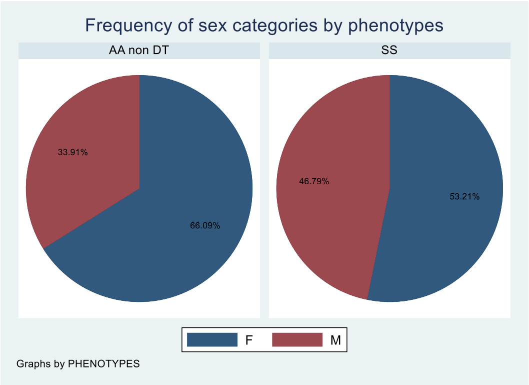
Frequency of sex categories by phenotypes
AA non DT
SS
33.91%
66.09%
46.79%
53.21%
F
M
Graphs by PHENOTYPES

. summarize ageyears BMIkgm_n sbpmmhg dbpmmhg ugcrgg_n uacrmgg_n upcrmgg_n usg_

> n gfr_n creatugl_n bunmgdl_n creatmgdl_n glycemiagl_n hbgdl_n, detail

AGE (years)

Percentiles		Smallest		
1%	5	4		
5%	8	4		
10%	10	4	Obs	312
25%	17	5	Sum of Wgt.	312
50%	24		Mean	26.68269
		Largest	Std. Dev.	14.44844
75%	32	69		
90%	49	70	Variance	208.7575
95%	58	71	Skewness	.9439906
99%	69	77	Kurtosis	3.740819

BMI (kg/m²)

Percentiles		Smallest		
1%	10.56285	0		
5%	13.20018	0		
10%	14.1146	10.56285	Obs	290
25%	16.89624	11.2367	Sum of Wgt.	290
50%	19.93319		Mean	21.23138
		Largest	Std. Dev.	7.0038
75%	24.22145	39.54523		
90%	28.89664	49.60938	Variance	49.05322
95%	33.33333	61.22449	Skewness	1.753236
99%	49.60938	63.26531	Kurtosis	11.0602

SBP (mmHg)

Percentiles	Smallest			
1%	9	8		
5%	9	9		
10%	10	9	Obs	201
25%	11	9	Sum of Wgt.	201
50%	11		Mean	11.42786
Largest	Std. Dev.	1.336421		
75%	12	14		
90%	13	15	Variance	1.78602
95%	14	15	Skewness	.3186903
99%	15	16	Kurtosis	3.250151

DBP (mmHg)

Percentiles	Smallest			
1%	5	4		
5%	6	5		
10%	6	5	Obs	201
25%	6	5	Sum of Wgt.	201
50%	7		Mean	7.094527
Largest	Std. Dev.	.9929853		
75%	8	9		
90%	8	10	Variance	.9860199
95%	8	10	Skewness	.1787037
99%	10	11	Kurtosis	4.130733

UGCR (g/g)

Percentiles Smallest

1% 0 0

5% 0 0

10% 0 0 Obs 327

25% 0 0 Sum of Wgt. 327

50% 0 Mean .0530973

Largest Std. Dev. .4555013

75% .0111732 .9345794

90% .0265306 1.315789 Variance .2074814

95% .045977 3.184713 Skewness 13.85904

99% .9345794 7.373272 Kurtosis 212.3613

UACR (mg/g)

Percentiles Smallest

1% 2.264541 1.284553

5% 2.902857 2.212634

10% 3.74145 2.217795 Obs 324

25% 5.977987 2.264541 Sum of Wgt. 324

50% 13.28253 Mean 24.73155

Largest Std. Dev. 35.67064

75% 27.94813 208.2902

90% 56.64122 209.7872 Variance 1272.394

95% 77.70492 233.7209 Skewness 4.133535

99% 208.2902 328.6486 Kurtosis 26.68751

UPCR (mg/g)

Percentiles Smallest

1% 11.22941 9.212985

5% 17.01247 10.35513

10% 21.30393 10.88977 Obs 323

25% 37.23641 11.22941 Sum of Wgt. 323

50% 69.10204 Mean 184.96

Largest Std. Dev. 365.3178

75% 167.3077 1920.784

90% 401.6279 2716.279 Variance 133457.1

95% 656.5217 2804.145 Skewness 4.856734

99% 1920.784 2957.838 Kurtosis 30.84439

USG

Percentiles Smallest

1% 1.005 1.001

5% 1.008 1.004

10% 1.01 1.005 Obs 307

25% 1.011 1.005 Sum of Wgt. 307

50% 1.015 Mean 1.016274

Largest Std. Dev. .0059009

75% 1.02 1.03

90% 1.025 1.03 Variance .0000348

95% 1.026 1.03 Skewness .3383956

99% 1.03 1.035 Kurtosis 2.559198

GFR

Percentiles Smallest

1% 31.65795 10.85409

5% 64.76591 20.45519

10% 73.62174 31.65795 Obs 286

25% 94.91754 34.89279 Sum of Wgt. 286

50% 123.0369 Mean 122.7038

Largest Std. Dev. 44.23484

75% 146.0028 241.192

90% 170.6647 265.972 Variance 1956.721

95% 186.2326 358.9923 Skewness 1.505499

99% 265.972 407.8375 Kurtosis 10.89663

Creat U (g/l)

Percentiles Smallest

1% .235 .155

5% .49 .185

10% .58 .221 Obs 326

25% .835 .235 Sum of Wgt. 326

50% 1.359 Mean 1.84592

Largest Std. Dev. 1.376243

75% 2.585 6.4535

90% 3.8505 6.785 Variance 1.894044

95% 4.383 8.54 Skewness 1.61467

99% 6.4535 8.86 Kurtosis 6.572314

BUN (mg/dl)

Percentiles Smallest

1% .03 .025

5% .04 .025

10% .05 .03 Obs 305

25% .06 .03 Sum of Wgt. 305

50% .075 Mean .080541

Largest Std. Dev. .0417399

75% .095 .21

90% .12 .25 Variance .0017422

95% .135 .315 Skewness 5.559692

99% .21 .545 Kurtosis 55.60227

Creat (mg/dl)

Percentiles Smallest

1% .23 .13

5% .31 .16

10% .362 .22 Obs 317

25% .52 .23 Sum of Wgt. 317

50% .67 Mean .7333312

Largest Std. Dev. .4822367

75% .86 1.945

90% 1.09 2.11 Variance .2325522

95% 1.27 2.12 Skewness 8.512614

99% 1.945 7.36 Kurtosis 114.1765

Glycemia (g/l)

Percentiles Smallest

1% .7 .6

5% .72 .62

10% .75 .66 Obs 322

25% .8 .7 Sum of Wgt. 322

50% .85 Mean .8533416

Largest Std. Dev. .0834919

75% .91 1.06

90% .96 1.08 Variance .0069709

95% .99 1.09 Skewness .1530377

99% 1.06 1.1 Kurtosis 3.020297

Hb (g/dl)

Percentiles Smallest

1% 5.9 5.1

5% 7 5.5

10% 7.5 5.7 Obs 318

25% 8.5 5.9 Sum of Wgt. 318

50% 11.2 Mean 11.12673

Largest Std. Dev. 2.914731

75% 13.5 16.9

90% 15.2 17 Variance 8.495656

95% 15.8 17.9 Skewness .1203603

99% 16.9 18.2 Kurtosis 1.913712

. by phenotypes, sort : summarize ageyears BMIkgm_n sbpmmhg dbpmmhg ugcrgg_n ua

> crmgg_n upcrmgg_n usg_n gfr_n creatugl_n bunmgdl_n creatmgdl_n glycemiagl_n

> hbgdl_n, detail

-> phenotypes = AA non DT

AGE (years)

Percentiles Smallest

1% 5 4

5% 10 5

10% 15 5 Obs 168

25% 22 8 Sum of Wgt. 168

50% 29 Mean 32.08929

Largest Std. Dev. 15.34546

75% 40 69

90% 57 70 Variance 235.483

95% 61 71 Skewness .7224162

99% 71 77 Kurtosis 3.001759

BMI (kg/m²)

Percentiles Smallest

1% 0 0

5% 13.51644 0

10% 15.427 10.56285 Obs 162

25% 18.87755 12.04411 Sum of Wgt. 162

50% 22.3847 Mean 22.92112

Largest Std. Dev. 6.754727

75% 26.72993 37.87755

90% 31.2213 38.30317 Variance 45.62634

95% 34.02035 39.54523 Skewness .1915183

99% 39.54523 49.60938 Kurtosis 4.875264

SBP (mmHg)

Percentiles Smallest

1% 9 9

5% 10 9

10% 10 9 Obs 100

25% 11 10 Sum of Wgt. 100

50% 12 Mean 11.7

Largest Std. Dev. 1.32954

75% 12.5 14

90% 13 15 Variance 1.767677

95% 14 15 Skewness .4069058

99% 15.5 16 Kurtosis 3.401567

DBP (mmHg)

Percentiles Smallest

1% 5 5

5% 6 5

10% 6 6 Obs 100

25% 7 6 Sum of Wgt. 100

50% 7 Mean 7.25

Largest Std. Dev. .8211227

75% 8 9

90% 8 9 Variance .6742424

95% 8 9 Skewness -.2681754

99% 9 9 Kurtosis 3.012036

UGCR (g/g)

Percentiles Smallest

1% 0 0

5% 0 0

10% 0 0 Obs 176

25% 0 0 Sum of Wgt. 176

50% 0 Mean .0043793

Largest Std. Dev. .0081416

75% .0067975 .0363636

90% .0128205 .0365059 Variance .0000663

95% .0200501 .0442804 Skewness 2.944001

99% .0442804 .0519481 Kurtosis 13.89307

UACR (mg/g)

Percentiles Smallest

1% 2.212634 1.284553

5% 2.619808 2.212634

10% 2.996755 2.217795 Obs 173

25% 4.194122 2.264541 Sum of Wgt. 173

50% 6.521739 Mean 10.58006

Largest Std. Dev. 13.87203

75% 12.16179 48.48485

90% 20.23468 48.67188 Variance 192.4332

95% 32.42604 69.20924 Skewness 5.578316

99% 69.20924 139.0698 Kurtosis 46.22677

UPCR (mg/g)

Percentiles Smallest

1% 10.35513 9.212985

5% 15.07112 10.35513

10% 17.44239 10.88977 Obs 173

25% 25.54288 11.22941 Sum of Wgt. 173

50% 44.74194 Mean 64.79121

Largest Std. Dev. 68.94344

75% 73.72668 297.485

90% 126.7181 309.2105 Variance 4753.198

95% 181.4847 401.6279 Skewness 3.399907

99% 401.6279 530.2738 Kurtosis 18.43167

USG

Percentiles Smallest

1% 1.007 1.004

5% 1.01 1.007

10% 1.012 1.009 Obs 154

25% 1.017 1.009 Sum of Wgt. 154

50% 1.02 Mean 1.020006

Largest Std. Dev. .0053192

75% 1.024 1.03

90% 1.026 1.03 Variance .0000283

95% 1.028 1.03 Skewness -.2985073

99% 1.03 1.035 Kurtosis 3.124557

GFR

Percentiles Smallest

1% 20.45519 10.85409

5% 68.27573 20.45519

10% 73.94323 31.65795 Obs 157

25% 88.91493 34.89279 Sum of Wgt. 157

50% 114.4124 Mean 111.7363

Largest Std. Dev. 32.06331

75% 134.0415 168.7913

90% 149.0465 186.2326 Variance 1028.056

95% 160.5992 198.4262 Skewness -.2215852

99% 198.4262 200.3695 Kurtosis 3.578274

Creat U (g/l)

Percentiles Smallest

1% .495 .221

5% .76 .495

10% .895 .526 Obs 174

25% 1.3795 .535 Sum of Wgt. 174

50% 2.271 Mean 2.51379

Largest Std. Dev. 1.446276

75% 3.44 5.8885

90% 4.33 6.4535 Variance 2.091714

95% 4.856 8.54 Skewness 1.18674

99% 8.54 8.86 Kurtosis 5.529523

BUN (mg/dl)

Percentiles Smallest

1% .03 .025

5% .05 .03

10% .055 .04 Obs 162

25% .065 .04 Sum of Wgt. 162

50% .08 Mean .0880864

Largest Std. Dev. .0344574

75% .105 .165

90% .125 .17 Variance .0011873

95% .14 .21 Skewness 2.24408

99% .21 .315 Kurtosis 13.98454

Creat (mg/dl)

Percentiles Smallest

1% .24 .23

5% .383 .24

10% .497 .247 Obs 166

25% .62 .25 Sum of Wgt. 166

50% .777 Mean .8609157

Largest Std. Dev. .6013865

75% .98 1.945

90% 1.23 2.11 Variance .3616657

95% 1.4 2.12 Skewness 7.824569

99% 2.12 7.36 Kurtosis 83.63363

Glycemia (g/l)

Percentiles Smallest

1% .7 .7

5% .73 .7

10% .76 .7 Obs 170

25% .8 .7 Sum of Wgt. 170

50% .84 Mean .8556471

Largest Std. Dev. .0775352

75% .91 1.02

90% .95 1.02 Variance .0060117

95% .98 1.09 Skewness .3215538

99% 1.09 1.1 Kurtosis 2.968592

Hb (g/dl)

Percentiles Smallest

1% 8 7.4

5% 10.2 8

10% 11.2 8.8 Obs 170

25% 12.4 9.1 Sum of Wgt. 170

50% 13.3 Mean 13.38294

Largest Std. Dev. 1.821763

75% 14.5 16.9

90% 15.8 17 Variance 3.31882

95% 16.2 17.9 Skewness -.2397847

99% 17.9 18.2 Kurtosis 3.551123

-> phenotypes = SS

AGE (years)

Percentiles Smallest

1% 4 4

5% 6 4

10% 8 5 Obs 144

25% 12 6 Sum of Wgt. 144

50% 20 Mean 20.375

Largest Std. Dev. 10.2274

75% 26 43

90% 34 45 Variance 104.5997

95% 38 49 Skewness .683576

99% 49 57 Kurtosis 3.408119

BMI (kg/m^2)

Percentiles Smallest

1% 11.96775 11.2367

5% 13.15193 11.96775

10% 13.55529 11.96775 Obs 128

25% 15.74317 12.3452 Sum of Wgt. 128

50% 18.00952 Mean 19.09281

Largest Std. Dev. 6.747755

75% 20.9527 33.33333

90% 24.00549 33.71488 Variance 45.53219

95% 26.03749 61.22449 Skewness 4.235867

99% 61.22449 63.26531 Kurtosis 27.16522

SBP (mmHg)

Percentiles Smallest

1% 9 8

5% 9 9

10% 10 9 Obs 101

25% 10 9 Sum of Wgt. 101

50% 11 Mean 11.15842

Largest Std. Dev. 1.294084

75% 12 14

90% 13 14 Variance 1.674653

95% 14 14 Skewness .2319202

99% 14 14 Kurtosis 2.95686

DBP (mmHg)

Percentiles Smallest

1% 5 4

5% 5 5

10% 6 5 Obs 101

25% 6 5 Sum of Wgt. 101

50% 7 Mean 6.940594

Largest Std. Dev. 1.120908

75% 8 9

90% 8 10 Variance 1.256436

95% 8 10 Skewness .5452782

99% 10 11 Kurtosis 4.436952

UGCR (g/g)

Percentiles Smallest

1% 0 0

5% 0 0

10% 0 0 Obs 151

25% 0 0 Sum of Wgt. 151

50% .0051813 Mean .1098812

Largest Std. Dev. .666947

75% .017094 .9345794

90% .045977 1.315789 Variance .4448183

95% .1935484 3.184713 Skewness 9.356007

99% 3.184713 7.373272 Kurtosis 97.53371

UACR (mg/g)

Percentiles Smallest

1% 4.465733 2.635659

5% 6.857143 4.465733

10% 9.325018 4.697987 Obs 151

25% 16.25468 4.958184 Sum of Wgt. 151

50% 25.66845 Mean 40.94486

Largest Std. Dev. 44.98485

75% 48.57143 208.2902

90% 77.70492 209.7872 Variance 2023.637

95% 122.6316 233.7209 Skewness 3.265169

99% 233.7209 328.6486 Kurtosis 16.9563

UPCR (mg/g)

Percentiles Smallest

1% 19.72973 17.24138

5% 29.40767 19.72973

10% 45.18859 20.42194 Obs 150

25% 70.74334 21.70213 Sum of Wgt. 150

50% 156.6704 Mean 323.5547

Largest Std. Dev. 496.8096

75% 303.5294 1920.784

90% 707.2093 2716.279 Variance 246819.8

95% 1388.767 2804.145 Skewness 3.341108

99% 2804.145 2957.838 Kurtosis 15.3316

USG

Percentiles Smallest

1% 1.005 1.001

5% 1.007 1.005

10% 1.009 1.005 Obs 153

25% 1.01 1.005 Sum of Wgt. 153

50% 1.012 Mean 1.012516

Largest Std. Dev. .0036457

75% 1.015 1.02

90% 1.015 1.021 Variance .0000133

95% 1.02 1.024 Skewness .3392769

99% 1.024 1.025 Kurtosis 4.216059

GFR

Percentiles Smallest

1% 47.36239 38.30725

5% 58.50642 47.36239

10% 69.3 51.625 Obs 129

25% 102.8286 55.70698 Sum of Wgt. 129

50% 136.9561 Mean 136.0517

Largest Std. Dev. 52.69461

75% 163.924 241.192

90% 184.0071 265.972 Variance 2776.722

95% 196.7909 358.9923 Skewness 1.562687

99% 358.9923 407.8375 Kurtosis 9.34806

Creat U (g/l)

Percentiles Smallest

1% .185 .155

5% .37 .185

10% .49 .235 Obs 152

25% .684 .25 Sum of Wgt. 152

50% .875 Mean 1.081385

Largest Std. Dev. .760092

75% 1.23225 3.26

90% 1.815 3.415 Variance .5777399

95% 2.33 3.565 Skewness 3.595538

99% 3.565 6.785 Kurtosis 23.74932

BUN (mg/dl)

Percentiles Smallest

1% .03 .025

5% .04 .03

10% .045 .035 Obs 143

25% .055 .04 Sum of Wgt. 143

50% .065 Mean .071993

Largest Std. Dev. .0473827

75% .08 .135

90% .1 .14 Variance .0022451

95% .105 .25 Skewness 7.42966

99% .25 .545 Kurtosis 71.97552

Creat (mg/dl)

Percentiles Smallest

1% .16 .13

5% .269 .16

10% .323 .22 Obs 151

25% .426 .25 Sum of Wgt. 151

50% .57 Mean .5930728

Largest Std. Dev. .232938

75% .73 1.14

90% .87 1.29 Variance .0542601

95% .99 1.38 Skewness 1.00046

99% 1.38 1.58 Kurtosis 5.055942

Glycemia (g/l)

Percentiles Smallest

1% .62 .6

5% .71 .62

10% .73 .66 Obs 152

25% .79 .7 Sum of Wgt. 152

50% .85 Mean .8507632

Largest Std. Dev. .0898773

75% .91 1.05

90% .97 1.05 Variance .0080779

95% 1.01 1.06 Skewness .0521707

99% 1.06 1.08 Kurtosis 2.930605

Hb (g/dl)

Percentiles Smallest

1% 5.5 5.1

5% 6.6 5.5

10% 7 5.7 Obs 148

25% 7.6 5.9 Sum of Wgt. 148

50% 8.4 Mean 8.535135

Largest Std. Dev. 1.362364

75% 9.3 11.6

90% 10.7 11.6 Variance 1.856036

95% 11 11.6 Skewness .3673008

99% 11.6 12 Kurtosis 2.951026

```
. by phenotypes, sort : summarize ageyears BMIkgm_n sbpmmhg dbpmmhg creatugl_n
> bunmgdl_n creatmgdl_n glycemiagl_n hbgdl_n

--------------------------------------------------------------------------------
-> phenotypes = AA non DT

    Variable |        Obs        Mean    Std. Dev.       Min        Max
-------------+---------------------------------------------------------
    ageyears |        168    32.08929    15.34546          4         77
    BMIkgm_n |        162    22.92112    6.754727          0   49.60938
     sbpmmhg |        100        11.7     1.32954          9         16
     dbpmmhg |        100        7.25    .8211227          5          9
  creatugl_n |        174     2.51379    1.446276       .221       8.86
-------------+---------------------------------------------------------
   bunmgdl_n |        162    .0880864    .0344574       .025       .315
creatmgdl_n |        166    .8609157    .6013865        .23       7.36
glycemiagl_n |        170    .8556471    .0775352         .7        1.1
     hbgdl_n |        170    13.38294    1.821763        7.4       18.2

--------------------------------------------------------------------------------
-> phenotypes = SS

    Variable |        Obs        Mean    Std. Dev.       Min        Max
-------------+---------------------------------------------------------
    ageyears |        144      20.375     10.2274          4         57
    BMIkgm_n |        128    19.09281    6.747755    11.2367   63.26531
     sbpmmhg |        101    11.15842    1.294084          8         14
     dbpmmhg |        101    6.940594    1.120908          4         11
  creatugl_n |        152    1.081385     .760092       .155      6.785
-------------+---------------------------------------------------------
   bunmgdl_n |        143     .071993    .0473827       .025       .545
creatmgdl_n |        151    .5930728     .232938        .13       1.58
glycemiagl_n |        152    .8507632    .0898773         .6       1.08
     hbgdl_n |        148    8.535135    1.362364        5.1         12

.
```

ranksum ageyears, by(phenotypes)

Two-sample Wilcoxon rank-sum (Mann-Whitney) test

. ranksum ageyears, by(phenotypes)

Two-sample Wilcoxon rank-sum (Mann-Whitney) test

phenotypes	obs	rank sum	expected
AA non DT	168	32047	26292
SS	144	16781	22536
combined	312	48828	48828

unadjusted variance 631008.00

adjustment for ties -549.25

adjusted variance 630458.75

Ho: ageyears(phenot~s==AA non DT) = ageyears(phenot~s==SS)

z = 7.248

Prob > z = 0.0000

. ranksum BMIkgm_n, by(phenotypes)

Two-sample Wilcoxon rank-sum (Mann-Whitney) test

phenotypes	obs	rank sum	expected
AA non DT	162	28218	23571
SS	128	13977	18624
combined	290	42195	42195

unadjusted variance 502848.00

adjustment for ties -3.83

adjusted variance 502844.17

Ho: BMIkgm_n(phenot~s==AA non DT) = BMIkgm_n(phenot~s==SS)

z = 6.553

Prob > z = 0.0000

. ranksum sbpmmhg, by(phenotypes)

Two-sample Wilcoxon rank-sum (Mann-Whitney) test

phenotypes	obs	rank sum	expected
AA non DT	100	11250	10100
SS	101	9051	10201
combined	201	20301	20301

unadjusted variance 170016.67

adjustment for ties -9423.15

adjusted variance 160593.52

Ho: sbpmmhg(phenot~s==AA non DT) = sbpmmhg(phenot~s==SS)

z = 2.870

Prob > z = 0.0041

. ranksum sbpmmhg, by(phenotypes)

Two-sample Wilcoxon rank-sum (Mann-Whitney) test

phenotypes	obs	rank sum	expected
AA non DT	100	11250	10100
SS	101	9051	10201
combined	201	20301	20301

unadjusted variance 170016.67

adjustment for ties -9423.15

adjusted variance 160593.52

Ho: sbpmmhg(phenot~s==AA non DT) = sbpmmhg(phenot~s==SS)

z = 2.870

Prob > z = 0.0041

. ranksum dbpmmhg, by(phenotypes)

Two-sample Wilcoxon rank-sum (Mann-Whitney) test

phenotypes	obs	rank sum	expected
AA non DT	100	11127.5	10100
SS	101	9173.5	10201
combined	201	20301	20301

unadjusted variance 170016.67

adjustment for ties -17618.97

adjusted variance 152397.69

Ho: dbpmmhg(phenot~s==AA non DT) = dbpmmhg(phenot~s==SS)

z = 2.632

Prob > z = 0.0085

. ranksum ugcrgg_n, by(phenotypes)

Two-sample Wilcoxon rank-sum (Mann-Whitney) test

phenotypes	obs	rank sum	expected
AA non DT	176	25399.5	28864
SS	151	28228.5	24764
combined	327	53628	53628

unadjusted variance 726410.67

adjustment for ties -117163.68

adjusted variance 609246.98

Ho: ugcrgg_n(phenot~s==AA non DT) = ugcrgg_n(phenot~s==SS)

z = -4.439

Prob > z = 0.0000

. ranksum uacrmgg_n, by(phenotypes)

Two-sample Wilcoxon rank-sum (Mann-Whitney) test

phenotypes	obs	rank sum	expected
AA non DT	173	18266	28112.5
SS	151	34384	24537.5
combined	324	52650	52650

unadjusted variance 707497.92

adjustment for ties -0.12

adjusted variance 707497.79

Ho: uacrmg~n(phenot~s==AA non DT) = uacrmg~n(phenot~s==SS)

z = -11.706

Prob > z = 0.0000

. ranksum upcrmgg_n, by(phenotypes)

Two-sample Wilcoxon rank-sum (Mann-Whitney) test

phenotypes	obs	rank sum	expected
AA non DT	173	19447	28026
SS	150	32879	24300
combined	323	52326	52326

unadjusted variance 700650.00

adjustment for ties 0.00

adjusted variance 700650.00

Ho: upcrmg~n(phenot~s==AA non DT) = upcrmg~n(phenot~s==SS)

z = -10.249

Prob > z = 0.0000

. ranksum usg_n, by(phenotypes)

Two-sample Wilcoxon rank-sum (Mann-Whitney) test

phenotypes	obs	rank sum	expected
AA non DT	154	32480	23716
SS	153	14798	23562
combined	307	47278	47278

unadjusted variance   604758.00

adjustment for ties   -4756.94

adjusted variance   600001.06

Ho: usg_n(phenot~s==AA non DT) = usg_n(phenot~s==SS)

z = 11.314

Prob > z =   0.0000

. ranksum gfr_n, by(phenotypes)

Two-sample Wilcoxon rank-sum (Mann-Whitney) test

phenotypes	obs	rank sum	expected
AA non DT	157	19370.5	22529.5
SS	129	21670.5	18511.5
combined	286	41041	41041

unadjusted variance   484384.25

adjustment for ties   -0.62

adjusted variance   484383.63

Ho: gfr_n(phenot~s==AA non DT) = gfr_n(phenot~s==SS)

z = -4.539

Prob > z =   0.0000

. ranksum creatugl_n, by(phenotypes)

Two-sample Wilcoxon rank-sum (Mann-Whitney) test

phenotypes obs rank sum expected

AA non DT 174 37568.5 28449

SS 152 15732.5 24852

combined 326 53301 53301

unadjusted variance 720708.00

adjustment for ties -9.49

adjusted variance 720698.51

Ho: creatu~n(phenot~s==AA non DT) = creatu~n(phenot~s==SS)

z = 10.742

Prob > z = 0.0000

. ranksum bunmgdl_n, by(phenotypes)

Two-sample Wilcoxon rank-sum (Mann-Whitney) test

phenotypes obs rank sum expected

AA non DT 162 29446.5 24786

SS 143 17218.5 21879

combined 305 46665 46665

unadjusted variance 590733.00

adjustment for ties -2732.22

adjusted variance 588000.78

Ho: bunmgd~n(phenot~s==AA non DT) = bunmgd~n(phenot~s==SS)

z = 6.078

Prob > z = 0.0000

. ranksum creatmgdl_n, by(phenotypes)

Two-sample Wilcoxon rank-sum (Mann-Whitney) test

phenotypes	obs	rank sum	expected
AA non DT	166	32277	26394
SS	151	18126	24009
combined	317	50403	50403

unadjusted variance 664249.00

adjustment for ties -85.70

adjusted variance 664163.30

Ho: creatm~n(phenot~s==AA non DT) = creatm~n(phenot~s==SS)

z = 7.219

Prob > z = 0.0000

. ranksum glycemiagl_n, by(phenotypes)

Two-sample Wilcoxon rank-sum (Mann-Whitney) test

phenotypes	obs	rank sum	expected
AA non DT	170	27766	27455
SS	152	24237	24548
combined	322	52003	52003

unadjusted variance 695526.67

adjustment for ties -1138.73

adjusted variance 694387.94

Ho: glycem~n(phenot~s==AA non DT) = glycem~n(phenot~s==SS)

z = 0.373

Prob > z = 0.7090

. ranksum hbgdl_n, by(phenotypes)

Two-sample Wilcoxon rank-sum (Mann-Whitney) test

phenotypes	obs	rank sum	expected
AA non DT	170	39132	27115
SS	148	11589	23606
combined	318	50721	50721

unadjusted variance 668836.67

adjustment for ties -151.13

adjusted variance 668685.54

Ho: hbgdl_n(phenot~s==AA non DT) = hbgdl_n(phenot~s==SS)

z = 14.696

Prob > z = 0.0000

```
. tabulate sex phenotypes, chi2 column row
```

Key
frequency<br>*row percentage*<br>*column percentage*

	PHENOTYPES		
SEX	AA non DT	SS	Total
F	115<br>58.08<br>66.09	83<br>41.92<br>53.21	198<br>100.00<br>60.00
M	59<br>44.70<br>33.91	73<br>55.30<br>46.79	132<br>100.00<br>40.00
Total	174<br>52.73<br>100.00	156<br>47.27<br>100.00	330<br>100.00<br>100.00

Pearson chi2(1) = 5.6917 Pr = 0.017

.

```
. tabulate ugcrmgg2g phenotypes, chi2 column row
```

Key
frequency
row percentage
column percentage

	PHENOTYPES		
UGCR(mg/g)2G	AA non DT	SS	Total
UGCR<0,020	167	117	284
	58.80	41.20	100.00
	94.89	77.48	86.85
UGCR≥0,020	9	34	43
	20.93	79.07	100.00
	5.11	22.52	13.15
Total	176	151	327
	53.82	46.18	100.00
	100.00	100.00	100.00

Pearson chi2(1) = 21.5524 Pr = 0.000

. tabulate UACRmgg03030□ 2G phenotypes, chi2 column row

Key
frequency<br>*row percentage*<br>*column percentage*

UACR (mg/g)]0; 30[[30;+□] 2G	PHENOTYPES AA non DT	SS	Total
UACR < 30	163<br>65.20<br>94.22	87<br>34.80<br>57.62	250<br>100.00<br>77.16
UACR ≥ 30	10<br>13.51<br>5.78	64<br>86.49<br>42.38	74<br>100.00<br>22.84
Total	173<br>53.40<br>100.00	151<br>46.60<br>100.00	324<br>100.00<br>100.00

Pearson chi2(1) = 61.2982 Pr = 0.000

```
. tabulate upcrmgg200mgg2g phenotypes, chi2 column row
```

Key
frequency
row percentage
column percentage

UPCR (mg/g) 200mg/g 2G	PHENOTYPES AA non DT	SS	Total
UPCR > 200	8	59	67
	11.94	88.06	100.00
	4.62	39.33	20.74
UPCR ≤ 200	165	91	256
	64.45	35.55	100.00
	95.38	60.67	79.26
Total	173	150	323
	53.56	46.44	100.00
	100.00	100.00	100.00

Pearson chi2(1) = 58.8723 Pr = 0.000

```
. tabulate glomerularproteinuriauacrupcrou5 phenotypes, chi2 column row

+-------------------+
| Key               |
|-------------------|
|     frequency     |
|  row percentage   |
| column percentage |
+-------------------+

  Glomerular |
 proteinuria |
(UACR/UPCR > |
 ou = 59 %); |
   physio ou |
 patho (UPCR |      PHENOTYPES
  <, =  ou > | AA non DT         SS |     Total
-------------+----------------------+----------
PHYSIOLOGICAL|         4          6 |        10 
             |     40.00      60.00 |    100.00 
             |    100.00     100.00 |    100.00 
-------------+----------------------+----------
       Total |         4          6 |        10 
             |     40.00      60.00 |    100.00 
             |    100.00     100.00 |    100.00 
```

```
. tabulate tubularproteinuriauacrupcr59phys phenotypes, chi2 column row

+-------------------+
| Key               |
|-------------------|
|     frequency     |
|  row percentage   |
| column percentage |
+-------------------+

     Tubular |
 proteinuria |
(UACR/UPCR < |
 59%; physio |
    ou patho |
(UPCR <, = ou|      PHENOTYPES
 > 200 mg/g) | AA non DT         SS |     Total
-------------+----------------------+----------
 PATHOLOGICAL|         8         59 |        67 
             |     11.94      88.06 |    100.00 
             |      4.73      40.97 |     21.41 
-------------+----------------------+----------
PHYSIOLOGICAL|       161         85 |       246 
             |     65.45      34.55 |    100.00 
             |     95.27      59.03 |     78.59 
-------------+----------------------+----------
       Total |       169        144 |       313 
             |     53.99      46.01 |    100.00 
             |    100.00     100.00 |    100.00 

          Pearson chi2(1) =  60.6909   Pr = 0.000
```

```
. tabulate usg10102g phenotypes, chi2 column row
```

Key
frequency
row percentage
column percentage

USG 1,010 2G	PHENOTYPES AA non DT	SS	Total
USG > 1,010	146	99	245
	59.59	40.41	100.00
	94.81	64.71	79.80
USG ≤ 1,010	8	54	62
	12.90	87.10	100.00
	5.19	35.29	20.20
Total	154	153	307
	50.16	49.84	100.00
	100.00	100.00	100.00

Pearson chi2(1) = 43.1426 Pr = 0.000

```
. tabulate gfr4g phenotypes, chi2 column row
```

Key
frequency
row percentage
column percentage

GFR 4G	PHENOTYPES AA non DT	SS	Total
GFR < 60	6	7	13
	46.15	53.85	100.00
	3.82	5.47	4.56
GFR > 140	31	61	92
	33.70	66.30	100.00
	19.75	47.66	32.28
GFR [60; 90[	35	14	49
	71.43	28.57	100.00
	22.29	10.94	17.19
GFR [90; 140]	85	46	131
	64.89	35.11	100.00
	54.14	35.94	45.96
Total	157	128	285
	55.09	44.91	100.00
	100.00	100.00	100.00

Pearson chi2(3) = 27.8073 Pr = 0.000

Odds ratio

UGCR

```
. cci 34 9 117 167
```

	Exposed	Unexposed	Total	Proportion Exposed	
Cases	34	9	43	0.7907	
Controls	117	167	284	0.4120	
Total	151	176	327	0.4618	
	Point estimate		[95% Conf.	Interval]	
Odds ratio	5.392213		2.407055	13.2154	(exact)
Attr. frac. ex.	.8145474		.5845546	.9243307	(exact)
Attr. frac. pop	.6440607				

chi2(1) = 21.55 Pr>chi2 = 0.0000

UACR

```
. cci 64 10 87 163
```

	Exposed	Unexposed	Total	Proportion Exposed	
Cases	64	10	74	0.8649	
Controls	87	163	250	0.3480	
Total	151	173	324	0.4660	
	Point estimate		[95% Conf.	Interval]	
Odds ratio	11.9908		5.707243	27.32816	(exact)
Attr. frac. ex.	.9166028		.824784	.9634077	(exact)
Attr. frac. pop	.7927375				

chi2(1) = 61.30 Pr>chi2 = 0.0000

UPCR

```
. cci 59 8 91 165
                                                         Proportion
                 |   Exposed   Unexposed  |      Total     Exposed
-----------------+------------------------+------------------------
           Cases |        59           8  |         67      0.8806
        Controls |        91         165  |        256      0.3555
-----------------+------------------------+------------------------
           Total |       150         173  |        323      0.4644
                 |                        |
                 |      Point estimate    |    [95% Conf. Interval]
                 |------------------------+------------------------
      Odds ratio |         13.37225       |    5.971085    33.58871 (exact)
 Attr. frac. ex. |         .9252183       |    .8325262    .9702281 (exact)
 Attr. frac. pop |         .8147445       |
                 +-------------------------------------------------
                               chi2(1) =    58.87  Pr>chi2 = 0.0000
```

Tubular proteinuria

```
. cci 59 8 85 161
                                                         Proportion
                 |   Exposed   Unexposed  |      Total     Exposed
-----------------+------------------------+------------------------
           Cases |        59           8  |         67      0.8806
        Controls |        85         161  |        246      0.3455
-----------------+------------------------+------------------------
           Total |       144         169  |        313      0.4601
                 |                        |
                 |      Point estimate    |    [95% Conf. Interval]
                 |------------------------+------------------------
      Odds ratio |         13.96912       |    6.216634    35.15126 (exact)
 Attr. frac. ex. |         .9284135       |    .8391412    .9715515 (exact)
 Attr. frac. pop |         .8175582       |
                 +-------------------------------------------------
                               chi2(1) =    60.69  Pr>chi2 = 0.0000
```

USG

. cci 54 8 99 146

	Exposed	Unexposed	Total	Proportion Exposed
Cases	54	8	62	0.8710
Controls	99	146	245	0.4041
Total	153	154	307	0.4984

	Point estimate	[95% Conf.	Interval]	
Odds ratio	9.954545	4.425615	25.11649	(exact)
Attr. frac. ex.	.8995434	.7740427	.9601855	(exact)
Attr. frac. pop	.7834733			

chi2(1) = 43.14 Pr>chi2 = 0.0000

.

GFR < 60 ml/min/1.73m^2

. cci 7 6 46 85

	Exposed	Unexposed	Total	Proportion Exposed
Cases	7	6	13	0.5385
Controls	46	85	131	0.3511
Total	53	91	144	0.3681

	Point estimate	[95% Conf.	Interval]	
Odds ratio	2.155797	.5787823	8.217766	(exact)
Attr. frac. ex.	.5361345	-.7277654	.8783124	(exact)
Attr. frac. pop	.2886878			

chi2(1) = 1.78 Pr>chi2 = 0.1817

60 ≤ GFR < 90 ml/min/1.73m^2

```
. cci 14 35 46 85
                                                         Proportion
                 |   Exposed   Unexposed  |      Total     Exposed
-----------------+------------------------+------------------------
           Cases |        14          35  |         49       0.2857
        Controls |        46          85  |        131       0.3511
-----------------+------------------------+------------------------
           Total |        60         120  |        180       0.3333
                 |                        |
                 |      Point estimate    |    [95% Conf. Interval]
                 |------------------------+------------------------
      Odds ratio |         .7391304       |    .3325266     1.58498 (exact)
 Prev. frac. ex. |         .2608696       |   -.5849803    .6674734 (exact)
 Prev. frac. pop |         .0916031       |
                 +-------------------------------------------------
                               chi2(1) =     0.69  Pr>chi2 = 0.4072
```

GFR ≥ 140 ml/min/1.73m^2

```
. cci 61 31 46 85
                                                         Proportion
                 |   Exposed   Unexposed  |      Total     Exposed
-----------------+------------------------+------------------------
           Cases |        61          31  |         92       0.6630
        Controls |        46          85  |        131       0.3511
-----------------+------------------------+------------------------
           Total |       107         116  |        223       0.4798
                 |                        |
                 |      Point estimate    |    [95% Conf. Interval]
                 |------------------------+------------------------
      Odds ratio |         3.636045       |    1.998956    6.640535 (exact)
 Attr. frac. ex. |         .7249759       |    .4997388    .8494097 (exact)
 Attr. frac. pop |         .4806905       |
                 +-------------------------------------------------
                               chi2(1) =    21.06  Pr>chi2 = 0.0000
```

SS (n = 163)

Variables	Median (min - max) or % (n)	5 – 95eme percentiles	observations
Anthropometric			
Age (years)	20 (4 - 57)	6 - 38	144
Female sex	53.21% (83)		156
BMI (kg/m²)	18 (11.24 – 33.71)	13.15 – 26.04	128
SBP (mmHg)	11 (8 - 14)	9 - 14	101
DBP (mmHg)	7 (4 - 11)	5 - 8	101
Biochemical			
Hb (g/dl)	8.4 (5.1 – 13.6)	6.6 - 11	148
Glucose Se, g/dl	85 (60 - 108)	71 - 101	152
BUN, mg/dl	6.5 (2.5 – 54.5)	4 – 10.5	143
Creatinin Se, mg/dl	0.57 (0.13 – 1.58)	0.27 – 0.99	151
Creatinine U, mg/dl	88 (16 - 679)	37 - 233	152
GFR, ml/min/1.73m²	136.96 (38.31 – 407.84)	58.51 -196.80	129
UACR, mg/g	25.67 (2.64 – 328.65)	6.86 – 122.63	151
UPCR, mg/g	156.67 (17.24 – 2957.84)	29.41 – 1388.76	150
UGCR, g/g	0.005 (0 – 7.37)	0 – 0.193	151
USG	1.012 (1.001 – 1.025)	1.007 - 1.020	153

AA non DT (n = 177)

Variables	Median (min – max)or % (n)	5 - 95eme percentiles	observations
Anthropometric			
Age (years)	29 (4 - 77)	10 - 61	168
Female sex	66.09% (115)		174
BMI (kg/m²)	22,38 (10.56 – 49.61)	13.52 – 34.02	162
SBP (mmHg)	12 (9 -16)	10 - 14	100
DBP (mmHg)	7 (5 - 9)	6 - 8	100
Biochemical			
Hb, g/dl	13.3 (7.4 – 18.2)	10.2 – 16.2	170
Glucose Se, g/dl	84 (70 - 110)	73 - 98	170
BUN, mg/dl	8 (2.5 – 31.5)	5 - 14	162
Créatinine Se, mg/dl	0.78 (0.23 – 7.36)	0.38 – 1.4	166
Créatinine U, mg/dl	227 (22 - 886)	76 - 486	174
GFR, ml/min/1.73m²	114.41 (10.85 – 200.37)	68.28 – 160.60	157
UACR, mg/g	6.52 (1.28 - 139.07)	2.62 – 32.43	173
UPCR, mg/g	44.74 (9.21 – 530.27)	15.07 - 181.48	173
UGCR, g/g	0 (0 – 0.052)	0 – 0.020	176
USG	1.02 (1.004 – 1.035)	1.010 – 1.028	154

	SS (163)	AA non DT (177)		SS (n = 45)	AA non DT (n = 45)	
Variables	M±SD ou %(n)	M±SD ou %(n)	p-value	M±SD ou %(n)	M±SD ou %(n)	p-value
Anthropometric						
Age (years)	20.375±10.227	32.089±15.345	< 0.001	21.38±10.36	21.38±10.36	1
Female sex	53.21%(83)	66.09%(115)		64.44%(29)	64.44%(29)	
Male sex	46.79%(73)	33.91% (59)	0.017	35.56%(16)	35.56%(16)	1
BMI (kg/m²)	19.09± 6.75	22.92±6.75	< 0.001	19,63±7,48	21.50±6,76	0.079
SBP (mmHg)	11.16±1,29	11.7±1.33	0.0041	11,23±1,16	11.82±1.01	0.076
DBP (mmHg)	6.94±1.12	7.25±0.82	0.0085	7,23±1,06	7,35±0,79	0.396
Biochemical						
Hb (g/dl)	8.57±1.47	13.38±1.82	< 0.001	8,69±1,43	13.05±1.87	< 0.001
Glucose Se, g/dl	85±9	86±8	0.709	85±7	86±10	0.447
BUN, mg/dl	7±4.5	9±3.5	< 0.001	6.5±2	8.5±2.5	0.0003
Créatinine Se, mg/dl	0.59±0.23	0.86±0.60	< 0.001	0.58±0.21	0.75±0.32	0.0095

	SS (n = 163)	AA non DT (n = 177)		
Variables	% (n)	% (n)	p-value	Odds ratio
USG≤ 1.010	35.29% (54)	5.19% (8)		
USG > 1.010	64.71% (99)	94.81% (146)	< 0.001	9.95 [4.43 – 25.12]
GFR ≥ 140 ml/min/1.73m^2	47,66% (61)	19,75% (31)	< 0.001	3.64 [2 – 6.64]
GFR < 60 ml/min/1.73m^2	5,47% (7)	3.82% (6)	0.182	
GFR 60; 90[ml/min/1.73m^2	10.94 (14)	22.29% (35)		
GFR 90; 140[ml/min/1.73m^2	35.66% (46)	54.14% (85)		
UACR ≥ 30 (mg/g)	42.38% (64)	5.78% (10)		
UACR < 30 (mg/g)	57.62% (87)	94.22% (163)		11.99 [5.71; 27.33]
UPCR > 200 (mg/g)	39.33% (59)	4.62% (8)		
UPCR ≤ 200 (mg/g)	60.67% (91)	95.38% (165)	< 0.001	13.37 [5.97; 33.59]
Presence glomerular PU	0	0		
Absence glomrular PU	6	4	NA	NA
Presence tubular PU	40.97% (59)	4.73% (8)		
Absence tubular PU	59.03% (85)	95.27% (161)	< 0.001	13.97 [6.22; 35.15]
UGCR ≥ 20 (mg/g)	22.52% (34)	5.11% (9)		
UGCR < 20 (g/g)	77.48% (117)	94.89% (167)	< 0.001	5.39 [2.41 – 13.21]

	SS (n = 45)	AA non DT (n = 45)		
Variables	% (n)	% (n)	p-value	Odds ratio
USG≤ 1.010	35.71% (15)	2.44 % (1)		
USG > 1.010	64.29% (27)	97.56% (40)	< 0.001	22.22 [2.98 - 958.10]
GFR ≥ 140 ml/min/1.73m^2	45.24% (19)	21.43% (9)	0.049	2.73 [0.89 – 8.61]
GFR < 60 ml/min/1.73m^2	2,38% (1)	2,38% (1)	0,859	
GFR [60; 90[ml/min/1.73m^2	11.90% (5)	23.81% (10)		
GFR [90; 140[ml/min/1.73m^2	40.48% (17)	52.38% (22)	0.118	
UACR ≥ 30 (mg/g)	47.73% (21)	8.89% (4)		
UACR < 30 (mg/g)	52.27% (23)	91.11% (41)	< 0.001	9.36 [2.64 – 41.02]
UPCR > 200 (mg/g)	37.21% (16)	2.22% (1)		
UPCR ≤ 200 (mg/g)	62.79% (27)	97.78% (44)	<0.001	26.07 [3.54 – 1117.37]
Presence glomerular PU	2	0		
Absence glomerular PU	0	0	NA	NA
Presence tubular PU	39.02% (16)	2.22% (1)		
Absence tubular PU	60.98% (25)	97.78% (44)	< 0.001	28.16 [3.80 – 1206.73]
UGCR ≥ 20 (mg/g)	25% (11)	6.67% (3)		
UGCR < 20 (mg/g)	75% (33)	93.33% (42)	0.018	4.67 [1.09 – 27.69]
