# Supplementary material for: Biomarkers of sickle cell nephropathy in Senegal
Source: PLoS One. 2022 Nov 21;17(11):e0273745. doi: 10.1371/journal.pone.0273745 (PMC9678278; doi:10.1371/journal.pone.0273745)
Supplement: S3 File — (PDF) [file pone.0273745.s003.pdf]

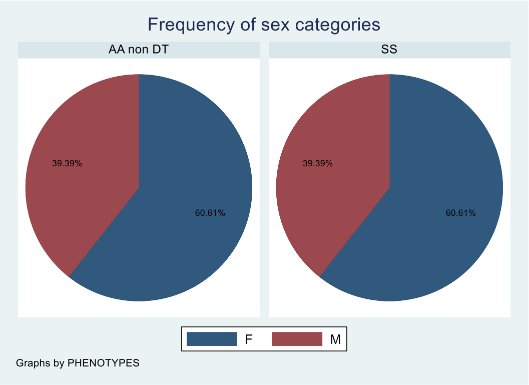

. by phenotypes, sort : summarize ageyears BMIkgm_n sbpmmhg dbpmmhg ugcrgg_n ua

> crmgg_n upcrmgg_n usg_n gfr_n creatugl_n bunmgdl_n creatmgdl_n glycemiagl_n h

> bgdl_n, detail

-> phenotypes = AA non DT

AGE (years)

Percentiles		Smallest		
1%	9	9		
5%	10	10		
10%	11	10	Obs	33
25%	18	11	Sum of Wgt.	33
50%	23		Mean	22.54545
Largest			Std. Dev.	8.008163

75% 28 33

90% 33 34 Variance 64.13068

95% 38 38 Skewness .0943275

99% 38 38 Kurtosis 2.278591

BMI (kg/m²)

Percentiles Smallest

1% 12.43512 12.43512

5% 13.51644 13.51644

10% 14.75797 14.71607 Obs 33

25% 18.25632 14.75797 Sum of Wgt. 33

50% 20.08507 Mean 20.39841

Largest Std. Dev. 3.836621

75% 22.22906 25.0995

90% 25.0995 25.71101 Variance 14.71966

95% 27.34375 27.34375 Skewness .0328783

99% 28.76397 28.76397 Kurtosis 2.728547

SBP (mmHg)

Percentiles Smallest

1% 10 10

5% 10 10

10% 10 10 Obs 18

25% 11 11 Sum of Wgt. 18

50% 11.5 Mean 11.5

Largest Std. Dev. 1.043185

75% 12 12

90% 13 12 Variance 1.088235

95% 14 13 Skewness .4798673

99% 14 14 Kurtosis 3.162162

DBP (mmHg)

Percentiles Smallest

1% 6 6

5% 6 6

10% 6 6 Obs 18

25% 7 7 Sum of Wgt. 18

50% 7 Mean 7.277778

Largest Std. Dev. .7519039

75% 8 8

90% 8 8 Variance .5653595

95% 8 8 Skewness -.4834188

99% 8 8 Kurtosis 1.990611

UGCR (g/g)

Percentiles Smallest

1% 0 0

5% 0 0

10% 0 0 Obs 33

25% 0 0 Sum of Wgt. 33

50% 0 Mean .0031708

Largest Std. Dev. .0058618

75% .0053677 .007874

90% .007874 .0111732 Variance .0000344

95% .0169823 .0169823 Skewness 2.454482

99% .0265306 .0265306 Kurtosis 9.331335

UACR (mg/g)

Percentiles Smallest

1% 2.264541 2.264541

5% 2.828077 2.828077

10% 3.960262 3.053645 Obs 33

25% 4.652908 3.960262 Sum of Wgt. 33

50% 6.540037 Mean 8.412291

Largest Std. Dev. 6.386419

75% 10.29653 15.67753

90% 15.67753 15.79863 Variance 40.78635

95% 15.83182 15.83182 Skewness 2.947096

99% 37.23676 37.23676 Kurtosis 13.66117

UPCR (mg/g)

Percentiles Smallest

1% 12.81172 12.81172

5% 14.09593 14.09593

10% 15.52824 15.32479 Obs 33

25% 23.92432 15.52824 Sum of Wgt. 33

50% 44.88189 Mean 50.27348

Largest Std. Dev. 37.55449

75% 56.31241 78.10909

90% 78.10909 84.64842 Variance 1410.34

95% 168.1685 168.1685 Skewness 2.141075

99% 179.7414 179.7414 Kurtosis 7.897913

USG

Percentiles Smallest

1% 1.009 1.009

5% 1.014 1.014

10% 1.0155 1.015 Obs 30

25% 1.019 1.016 Sum of Wgt. 30

50% 1.021 Mean 1.021433

Largest Std. Dev. .0050629

75% 1.025 1.028

90% 1.029 1.03 Variance .0000256

95% 1.03 1.03 Skewness -.1156152

99% 1.03 1.03 Kurtosis 2.805458

GFR

Percentiles Smallest

1% 10.85409 10.85409

5% 69.10343 69.10343

10% 71.64286 70.44782 Obs 31

25% 84.58118 71.64286 Sum of Wgt. 31

50% 118.9363 Mean 111.4806

Largest Std. Dev. 34.25112

75% 131.9849 151.8054

90% 151.8054 151.8266 Variance 1173.139

95% 156.0566 156.0566 Skewness -.5373998

99% 186.2326 186.2326 Kurtosis 3.966406

Creat U (g/l)

Percentiles Smallest

1% .221 .221

5% .895 .895

10% 1.375 1.27 Obs 33

25% 1.7655 1.375 Sum of Wgt. 33

50% 2.32 Mean 2.685621

Largest Std. Dev. 1.37534

75% 3.6505 4.519

90% 4.519 4.9 Variance 1.891559

95% 5.8025 5.8025 Skewness .6459609

99% 5.8885 5.8885 Kurtosis 2.829917

BUN (mg/dl)

Percentiles Smallest

1% .04 .04

5% .04 .04

10% .0525 .05 Obs 30

25% .06 .055 Sum of Wgt. 30

50% .0775 Mean .092

Largest Std. Dev. .0519881

75% .12 .125

90% .13 .135 Variance .0027028

95% .155 .155 Skewness 2.715881

99% .315 .315 Kurtosis 12.32795

Creat (mg/dl)

Percentiles Smallest

1% .302 .302

5% .37 .37

10% .54 .528 Obs 31

25% .63 .54 Sum of Wgt. 31

50% .764 Mean .9789355

Largest Std. Dev. 1.209671

75% .916 1.016

90% 1.016 1.23 Variance 1.463304

95% 1.592 1.592 Skewness 4.955128

99% 7.36 7.36 Kurtosis 26.68203

Glycemia (g/l)

Percentiles Smallest

1% .71 .71

5% .78 .78

10% .8 .79 Obs 32

25% .825 .8 Sum of Wgt. 32

50% .86 Mean .8671875

Largest Std. Dev. .0645198

75% .915 .94

90% .94 .97 Variance .0041628

95% .97 .97 Skewness .090122

99% 1.02 1.02 Kurtosis 3.108538

Hb (g/dl)

Percentiles Smallest

1% 8 8

5% 8.8 8.8

10% 11 10.5 Obs 33

25% 12.5 11 Sum of Wgt. 33

50% 13.4 Mean 13.42121

Largest Std. Dev. 2.067268

75% 14.7 15.8

90% 15.8 15.8 Variance 4.273598

95% 16.8 16.8 Skewness -.3889819

99% 18.2 18.2 Kurtosis 3.89207

-> phenotypes = SS

AGE (years)

Percentiles Smallest

1% 9 9

5% 10 10

10% 11 10 Obs 33

25% 18 11 Sum of Wgt. 33

50% 23 Mean 22.54545

Largest Std. Dev. 8.008163

75% 28 33

90% 33 34 Variance 64.13068

95% 38 38 Skewness .0943275

99% 38 38 Kurtosis 2.278591

BMI (kg/m²)

Percentiles		Smallest		
1%	13.41713	13.41713		
5%	14.10911	14.10911		
10%	15.95046	15.15063	Obs	32
25%	17.01145	15.95046	Sum of Wgt.	32
50%	19.30129		Mean	19.26732
		Largest	Std. Dev.	3.157103
75%	20.89646	22.59814		
90%	22.59814	24.60938	Variance	9.967297
95%	26.03749	26.03749	Skewness	.4985016
99%	27.0538	27.0538	Kurtosis	3.159224

SBP (mmHg)

Percentiles		Smallest		
1%	9	9		
5%	9	9		
10%	10	10	Obs	25
25%	10	10	Sum of Wgt.	25
50%	11		Mean	11.24
		Largest	Std. Dev.	1.3
75%	12	12		
90%	13	13	Variance	1.69
95%	14	14	Skewness	.3571476
99%	14	14	Kurtosis	2.890171

DBP (mmHg)

Percentiles	Smallest			
1%	6	6		
5%	6	6		
10%	6	6	Obs	25
25%	6	6	Sum of Wgt.	25
50%	7		Mean	7.28
Largest	Std. Dev.	1.173314		
75%	8	8		
90%	8	8	Variance	1.376667
95%	9	9	Skewness	1.176777
99%	11	11	Kurtosis	5.06675

UGCR (g/g)

Percentiles	Smallest			
1%	0	0		
5%	0	0		
10%	0	0	Obs	33
25%	0	0	Sum of Wgt.	33
50%	0		Mean	.0103467
Largest	Std. Dev.	.0160249		
75%	.0128755	.04329		
90%	.04329	.0434783	Variance	.0002568
95%	.045977	.045977	Skewness	1.419197
99%	.0502513	.0502513	Kurtosis	3.581099

UACR (mg/g)

Percentiles Smallest

1% 4.697987 4.697987

5% 7.036011 7.036011

10% 8.887545 7.557604 Obs 33

25% 13.56725 8.887545 Sum of Wgt. 33

50% 29.95671 Mean 45.83054

Largest Std. Dev. 53.52857

75% 46.66667 106.1943

90% 106.1943 127.4227 Variance 2865.308

95% 208.2902 208.2902 Skewness 2.309506

99% 233.7209 233.7209 Kurtosis 7.937022

UPCR (mg/g)

Percentiles Smallest

1% 22.80702 22.80702

5% 28.73563 28.73563

10% 42.68456 34.48276 Obs 33

25% 67.2956 42.68456 Sum of Wgt. 33

50% 127.9592 Mean 305.9612

Largest Std. Dev. 645.4784

75% 193.7736 452.2156

90% 452.2156 623.0303 Variance 416642.4

95% 2716.279 2716.279 Skewness 3.464901

99% 2804.145 2804.145 Kurtosis 13.51046

USG

Percentiles Smallest

1% 1.001 1.001

5% 1.006 1.006

10% 1.01 1.01 Obs 33

25% 1.01 1.01 Sum of Wgt. 33

50% 1.012 Mean 1.012

Largest Std. Dev. .0036486

75% 1.015 1.016

90% 1.016 1.017 Variance .0000133

95% 1.02 1.02 Skewness -.1881634

99% 1.02 1.02 Kurtosis 4.659889

GFR

Percentiles Smallest

1% 94.91754 94.91754

5% 102.8286 102.8286

10% 114.2953 103.4025 Obs 33

25% 131.0035 114.2953 Sum of Wgt. 33

50% 154.0836 Mean 156.7991

Largest Std. Dev. 34.87621

75% 177.4106 196.7909

90% 196.7909 202.1034 Variance 1216.35

95% 226.4839 226.4839 Skewness .3342351

99% 241.192 241.192 Kurtosis 2.755685

Creat U (g/l)

Percentiles Smallest

1% .485 .485

5% .49 .49

10% .53 .494 Obs 33

25% .795 .53 Sum of Wgt. 33

50% .995 Mean 1.083879

Largest Std. Dev. .4497871

75% 1.2405 1.63

90% 1.63 1.805 Variance .2023084

95% 2.175 2.175 Skewness 1.021337

99% 2.33 2.33 Kurtosis 3.815897

BUN (mg/dl)

Percentiles Smallest

1% .03 .03

5% .04 .04

10% .04 .04 Obs 33

25% .05 .04 Sum of Wgt. 33

50% .06 Mean .0704545

Largest Std. Dev. .0385552

75% .08 .105

90% .105 .105 Variance .0014865

95% .105 .105 Skewness 3.1657

99% .25 .25 Kurtosis 15.36144

Creat (mg/dl)

Percentiles		Smallest		
1%	.25	.25		
5%	.25	.25		
10%	.31	.31	Obs	33
25%	.358	.31	Sum of Wgt.	33
50%	.49		Mean	.4934848
		Largest	Std. Dev.	.1711808
75%	.57	.77		
90%	.77	.8	Variance	.0293029
95%	.88	.88	Skewness	.7366546
99%	.88	.88	Kurtosis	2.841606

Glycemia (g/l)

Percentiles		Smallest		
1%	.71	.71		
5%	.72	.72		
10%	.77	.76	Obs	33
25%	.79	.77	Sum of Wgt.	33
50%	.85		Mean	.8487879
		Largest	Std. Dev.	.0761067
75%	.89	.94		
90%	.94	.97	Variance	.0057922
95%	1.01	1.01	Skewness	.3125784
99%	1.01	1.01	Kurtosis	2.551114

Hb (g/dl)

Percentiles	Smallest			
1%	6.6	6.6		
5%	6.7	6.7		
10%	6.8	6.8	Obs	29
25%	7.4	6.8	Sum of Wgt.	29
50%	8.3		Mean	8.337931
Largest			Std. Dev.	1.142183
75%	8.9	9.5		
90%	10	10	Variance	1.304581
95%	10.4	10.4	Skewness	.3639535
99%	11	11	Kurtosis	2.495134

```
. by phenotypes, sort : summarize ageyears BMIkgm_n sbpmmhg dbpmmhg creatugl_n
> bunmgdl_n creatmgdl_n glycemiagl_n hbgdl_n
```

-> phenotypes = AA non DT

Variable	Obs	Mean	Std. Dev.	Min	Max
ageyears	33	22.54545	8.008163	9	38
BMIkgm_n	33	20.39841	3.836621	12.43512	28.76397
sbpmmhg	18	11.5	1.043185	10	14
dbpmmhg	18	7.277778	.7519039	6	8
creatugl_n	33	2.685621	1.37534	.221	5.8885
bunmgdl_n	30	.092	.0519881	.04	.315
creatmgdl_n	31	.9789355	1.209671	.302	7.36
glycemiagl_n	32	.8671875	.0645198	.71	1.02
hbgdl_n	33	13.42121	2.067268	8	18.2

-> phenotypes = SS

Variable	Obs	Mean	Std. Dev.	Min	Max
ageyears	33	22.54545	8.008163	9	38
BMIkgm_n	32	19.26732	3.157103	13.41713	27.0538
sbpmmhg	25	11.24	1.3	9	14
dbpmmhg	25	7.28	1.173314	6	11
creatugl_n	33	1.083879	.4497871	.485	2.33
bunmgdl_n	33	.0704545	.0385552	.03	.25
creatmgdl_n	33	.4934848	.1711808	.25	.88
glycemiagl_n	33	.8487879	.0761067	.71	1.01
hbgdl_n	29	8.337931	1.142183	6.6	11

.

. ranksum ageyears, by(phenotypes)

Two-sample Wilcoxon rank-sum (Mann-Whitney) test

phenotypes	obs	rank sum	expected
AA non DT	33	1105.5	1105.5
SS	33	1105.5	1105.5

combined 66 2211 2211

unadjusted variance 6080.25
adjusted for ties -23.48

adjusted variance 6056.77

Ho: ageyears(phenot~s==AA non DT) = ageyears(phenot~s==SS)
z = 0.000
Prob > z = 1.0000

. ranksum BMIkgm_n, by(phenotypes)

Two-sample Wilcoxon rank-sum (Mann-Whitney) test

phenotypes	obs	rank sum	expected
AA non DT	33	1196	1089
SS	32	949	1056
combined	65	2145	2145

unadjusted variance 5808.00
adjustment for ties -0.13

adjusted variance 5807.87

Ho: BMIkgm_n(phenot~s==AA non DT) = BMIkgm_n(phenot~s==SS)
z = 1.404
Prob > z = 0.1603

. ranksum sbpmmhg, by(phenotypes)

Two-sample Wilcoxon rank-sum (Mann-Whitney) test

phenotypes	obs	rank sum	expected
AA non DT	18	426.5	396
SS	25	519.5	550
combined	43	946	946

unadjusted variance 1650.00

adjustment for ties -124.58

adjusted variance 1525.42

Ho: sbpmmhg(phenot~s==AA non DT) = sbpmmhg(phenot~s==SS)

z = 0.781

Prob > z = 0.4349

. ranksum dbpmmhg, by(phenotypes)

Two-sample Wilcoxon rank-sum (Mann-Whitney) test

phenotypes	obs	rank sum	expected
AA non DT	18	410.5	396
SS	25	535.5	550
combined	43	946	946

unadjusted variance 1650.00

adjustment for ties -175.04

adjusted variance 1474.96

Ho: dbpmmhg(phenot~s==AA non DT) = dbpmmhg(phenot~s==SS)

z = 0.378

Prob > z = 0.7058

. ranksum creatugl_n, by(phenotypes)

Two-sample Wilcoxon rank-sum (Mann-Whitney) test

phenotypes	obs	rank sum	expected
AA non DT	33	1547	1105.5
SS	33	664	1105.5
combined	66	2211	2211

unadjusted variance 6080.25

adjustment for ties -0.13

adjusted variance 6080.12

Ho: creatu~n(phenot~s==AA non DT) = creatu~n(phenot~s==SS)

z = 5.662

Prob > z = 0.0000

. ranksum bunmgdl_n, by(phenotypes)

Two-sample Wilcoxon rank-sum (Mann-Whitney) test

phenotypes	obs	rank sum	expected
AA non DT	30	1134	960
SS	33	882	1056
combined	63	2016	2016

unadjusted variance 5280.00

adjustment for ties -37.76

adjusted variance 5242.24

Ho: bunmgd~n(phenot~s==AA non DT) = bunmgd~n(phenot~s==SS)

z = 2.403

Prob > z = 0.0163

. ranksum creatmgdl_n, by(phenotypes)

Two-sample Wilcoxon rank-sum (Mann-Whitney) test

phenotypes	obs	rank sum	expected
AA non DT	31	1350	1007.5
SS	33	730	1072.5
combined	64	2080	2080

unadjusted variance 5541.25

adjustment for ties -3.81

adjusted variance 5537.44

Ho: creatm~n(phenot~s==AA non DT) = creatm~n(phenot~s==SS)

z = 4.603

Prob > z = 0.0000

. ranksum glycemiagl_n, by(phenotypes)

Two-sample Wilcoxon rank-sum (Mann-Whitney) test

phenotypes	obs	rank sum	expected
AA non DT	32	1143.5	1056
SS	33	1001.5	1089
combined	65	2145	2145

unadjusted variance 5808.00

adjustment for ties -19.04

adjusted variance 5788.96

Ho: glycem~n(phenot~s==AA non DT) = glycem~n(phenot~s==SS)

z = 1.150

Prob > z = 0.2501

. ranksum hbgdl_n, by(phenotypes)

Two-sample Wilcoxon rank-sum (Mann-Whitney) test

phenotypes	obs	rank sum	expected
AA non DT	33	1488.5	1039.5
SS	29	464.5	913.5
combined	62	1953	1953

unadjusted variance 5024.25

adjustment for ties -3.04

adjusted variance 5021.21

Ho: hbgdl_n(phenot~s==AA non DT) = hbgdl_n(phenot~s==SS)

z = 6.336

Prob > z = 0.0000

```
. tabulate sex phenotypes, chi2 column row
```

Key
frequency
row percentage
column percentage

SEX	PHENOTYPES AA non DT	SS	Total
F	20	20	40
	50.00	50.00	100.00
	60.61	60.61	60.61
M	13	13	26
	50.00	50.00	100.00
	39.39	39.39	39.39
Total	33	33	66
	50.00	50.00	100.00
	100.00	100.00	100.00

Pearson chi2(1) =   0.0000   Pr = 1.000

```
. tabulate ugcrgg2g phenotypes, chi2 column row
```

Key
frequency
row percentage
column percentage

UGCR(g/g)2G	PHENOTYPES AA non DT	SS	Total
UGCR<0,020	32	26	58
	55.17	44.83	100.00
	96.97	78.79	87.88
UGCR≥0,020	1	7	8
	12.50	87.50	100.00
	3.03	21.21	12.12
Total	33	33	66
	50.00	50.00	100.00
	100.00	100.00	100.00

Pearson chi2(1) = 5.1207 Pr = 0.024

. tabulate UACRmgg03030□ 2G phenotypes, chi2 column row

Key
frequency<br>*row percentage*<br>*column percentage*

UACR (mg/g)]0; 30[[30;+□] 2G	PHENOTYPES AA non DT	SS	Total
UACR < 30	32<br>65.31<br>96.97	17<br>34.69<br>51.52	49<br>100.00<br>74.24
UACR ≥ 30	1<br>5.88<br>3.03	16<br>94.12<br>48.48	17<br>100.00<br>25.76
Total	33<br>50.00<br>100.00	33<br>50.00<br>100.00	66<br>100.00<br>100.00

Pearson chi2(1) = 17.8271 Pr = 0.000

. tabulate upcrmgg200mgg2g phenotypes, chi2 column row

Key
frequency<br>*row percentage*<br>*column percentage*

UPCR (mg/g) 200mg/g 2G	PHENOTYPES AA non DT	SS	Total
UPCR > 200	0<br>0.00<br>0.00	7<br>100.00<br>21.21	7<br>100.00<br>10.61
UPCR ≤ 200	33<br>55.93<br>100.00	26<br>44.07<br>78.79	59<br>100.00<br>89.39
Total	33<br>50.00<br>100.00	33<br>50.00<br>100.00	66<br>100.00<br>100.00

Pearson chi2(1) = 7.8305 Pr = 0.005

```
. tabulate glomerularproteinuriauacrupcrou5 phenotypes, chi2 column row
```

Key
frequency
row percentage
column percentage

Glomerular proteinuria (UACR/UPCR > ou = 59 %); physio ou patho (UPCR <, = ou >	PHENOTYPES SS	Total
PHYSIOLOGICAL	3<br>100.00<br>100.00	3<br>100.00<br>100.00
Total	3<br>100.00<br>100.00	3<br>100.00<br>100.00

```
. tabulate tubularproteinuriauacrupcr59phys phenotypes, chi2 column row
```

Key
frequency
row percentage
column percentage

Tubular proteinuria (UACR/UPCR < 59%; physio ou patho (UPCR <, = ou > 200 mg/g)	PHENOTYPES AA non DT	SS	Total
PATHOLOGICAL	0<br>0.00<br>0.00	7<br>100.00<br>23.33	7<br>100.00<br>11.11
PHYSIOLOGICAL	33<br>58.93<br>100.00	23<br>41.07<br>76.67	56<br>100.00<br>88.89
Total	33<br>52.38<br>100.00	30<br>47.62<br>100.00	63<br>100.00<br>100.00

Pearson chi2(1) = 8.6625 Pr = 0.003

```
. tabulate usg10102g phenotypes, chi2 column row
```

Key
frequency
row percentage
column percentage

USG 1,010 2G	PHENOTYPES AA non DT	SS	Total
USG > 1,010	29	19	48
	60.42	39.58	100.00
	96.67	57.58	76.19
USG ≤ 1,010	1	14	15
	6.67	93.33	100.00
	3.33	42.42	23.81
Total	30	33	63
	47.62	52.38	100.00
	100.00	100.00	100.00

Pearson chi2(1) = 13.2372 Pr = 0.000

```
. tabulate gfr4g phenotypes, chi2 column row
```

Key
frequency
row percentage
column percentage

GFR 4G	PHENOTYPES AA non DT	SS	Total
GFR < 60	1	0	1
	100.00	0.00	100.00
	3.23	0.00	1.56
GFR > 140	5	22	27
	18.52	81.48	100.00
	16.13	66.67	42.19
GFR [60; 90[	7	0	7
	100.00	0.00	100.00
	22.58	0.00	10.94
GFR [90; 140]	18	11	29
	62.07	37.93	100.00
	58.06	33.33	45.31
Total	31	33	64
	48.44	51.56	100.00
	100.00	100.00	100.00

Pearson chi2(3) = 20.3507 Pr = 0.000

.

Odds ratio

UGCR

```
. cci 7 1 26 32
```

	Exposed	Unexposed	Total	Proportion Exposed	
Cases	7	1	8	0.8750	
Controls	26	32	58	0.4483	
Total	33	33	66	0.5000	
	Point estimate		[95% Conf. Interval]		
Odds ratio	8.615385		.9759941	400.0794	(exact)
Attr. frac. ex.	.8839286		-.0245963	.9975005	(exact)
Attr. frac. pop	.7734375				

chi2(1) = 5.12 Pr>chi2 = 0.0236

UACR

```
. cci 16 1 17 32
```

	Exposed	Unexposed	Total	Proportion Exposed	
Cases	16	1	17	0.9412	
Controls	17	32	49	0.3469	
Total	33	33	66	0.5000	
	Point estimate		[95% Conf. Interval]		
Odds ratio	30.11765		3.845026	1301.021	(exact)
Attr. frac. ex.	.9667969		.7399238	.9992314	(exact)
Attr. frac. pop	.9099265				

chi2(1) = 17.83 Pr>chi2 = 0.0000

UPCR

```
. cci 7 0 26 33
```

	Exposed	Unexposed	Total	Proportion Exposed	
Cases	7	0	7	1.0000	
Controls	26	33	59	0.4407	
Total	33	33	66	0.5000	
	Point estimate		[95% Conf. Interval]		
Odds ratio		.	2.181938	.	(Cornfield)
Attr. frac. ex.		.	.5416918	.	(Cornfield)
Attr. frac. pop		.			

chi2(1) = 7.83 Pr>chi2 = 0.0051

Odds ratio (UPCR) with correction adding 0.5 in each cell

```
. cci 15 1 53 67
```

	Exposed	Unexposed	Total	Proportion Exposed	
Cases	15	1	16	0.9375	
Controls	53	67	120	0.4417	
Total	68	68	136	0.5000	
	Point estimate		[95% Conf. Interval]		
Odds ratio	18.96226		2.705018	809.7447	(exact)
Attr. frac. ex.	.9472637		.6303168	.998765	(exact)
Attr. frac. pop	.8880597				

chi2(1) = 13.88 Pr>chi2 = 0.0002

Tubular proteinuria

```
. cci 7 0 23 33

                                                         Proportion
                 |   Exposed   Unexposed  |      Total     Exposed
-----------------+------------------------+------------------------
           Cases |         7           0  |          7      1.0000
        Controls |        23          33  |         56      0.4107
-----------------+------------------------+------------------------
           Total |        30          33  |         63      0.4762
                 |                        |
                 |      Point estimate    |    [95% Conf. Interval]
                 |------------------------+------------------------
      Odds ratio |                 .      |   2.455869           .  (Cornfield)
 Attr. frac. ex. |                 .      |   .5928121           .  (Cornfield)
 Attr. frac. pop |                 .      |
                 +-------------------------------------------------
                               chi2(1) =     8.66  Pr>chi2 = 0.0032
```

Odds ratio (tubular proteinuria) with correction adding 0.5 in each cell

```
. cci 15 1 47 67
                                                         Proportion
                 |   Exposed   Unexposed  |      Total     Exposed
-----------------+------------------------+------------------------
           Cases |        15           1  |         16      0.9375
        Controls |        47          67  |        114      0.4123
-----------------+------------------------+------------------------
           Total |        62          68  |        130      0.4769
                 |                        |
                 |      Point estimate    |    [95% Conf. Interval]
                 |------------------------+------------------------
      Odds ratio |         21.38298       |   3.032232    913.0947  (exact)
 Attr. frac. ex. |         .9532338       |     .67021    .9989048  (exact)
 Attr. frac. pop |         .8936567       |
                 +-------------------------------------------------
                               chi2(1) =    15.51  Pr>chi2 = 0.0001

.
```

USG

```
. cci 14 1 19 29
                                                         Proportion
                 |   Exposed   Unexposed  |      Total     Exposed
-----------------+------------------------+------------------------
           Cases |        14           1  |         15      0.9333
        Controls |        19          29  |         48      0.3958
-----------------+------------------------+------------------------
           Total |        33          30  |         63      0.5238
                 |                        |
                 |      Point estimate    |    [95% Conf. Interval]
                 |------------------------+------------------------
      Odds ratio |         21.36842       |    2.702454    933.6841 (exact)
 Attr. frac. ex. |          .953202       |    .6299659     .998929 (exact)
 Attr. frac. pop |         .8896552       |
                 +-------------------------------------------------
                               chi2(1) =    13.24  Pr>chi2 = 0.0003
```

GFR < 60 ml/min/1.73m^2

```
. cci 0 1 11 18

                                                         Proportion
                 |   Exposed   Unexposed  |      Total     Exposed
-----------------+------------------------+------------------------
           Cases |         0           1  |          1      0.0000
        Controls |        11          18  |         29      0.3793
-----------------+------------------------+------------------------
           Total |        11          19  |         30      0.3667
                 |                        |
                 |      Point estimate    |    [95% Conf. Interval]
                 |------------------------+------------------------
      Odds ratio |                0       |           0           . (Cornfield)
 Prev. frac. ex. |                1       |           .           1 (Cornfield)
 Prev. frac. pop |                .       |
                 +-------------------------------------------------
                               chi2(1) =     0.60  Pr>chi2 = 0.4390
```

Odds ratio (GFR < 60 ml/min/1.73m^2) with correction adding 0.5 in each cell

```
. cci 1 3 23 37
```

	Exposed	Unexposed	Total	Proportion Exposed	
Cases	1	3	4	0.2500	
Controls	23	37	60	0.3833	
Total	24	40	64	0.3750	
	Point estimate		[95% Conf. Interval]		
Odds ratio	.5362319		.0097951	7.209443	(exact)
Prev. frac. ex.	.4637681		-6.209443	.9902049	(exact)
Prev. frac. pop	.1777778				

chi2(1) = 0.28 Pr>chi2 = 0.5938

60 ≤ GFR < 90 ml/min/1.73m^2

```
. cci 7 0 11 18
```

	Exposed	Unexposed	Total	Proportion Exposed	
Cases	7	0	7	1.0000	
Controls	11	18	29	0.3793	
Total	18	18	36	0.5000	
	Point estimate		[95% Conf. Interval]		
Odds ratio	.		2.642669	.	(Cornfield)
Attr. frac. ex.	.		.6215947	.	(Cornfield)
Attr. frac. pop	.				

chi2(1) = 8.69 Pr>chi2 = 0.0032

Odds ratio (GFR < 60 ml/min/1.73m^2) with correction adding 0.5 in each cell

```
. cci 15 1 47 67
                                                         Proportion
                 |   Exposed   Unexposed  |      Total     Exposed
-----------------+------------------------+------------------------
           Cases |        15           1  |         16      0.9375
        Controls |        47          67  |        114      0.4123
-----------------+------------------------+------------------------
           Total |        62          68  |        130      0.4769
                 |                        |
                 |      Point estimate    |    [95% Conf. Interval]
                 |------------------------+------------------------
      Odds ratio |        21.38298        |   3.032232    913.0947 (exact)
 Attr. frac. ex. |        .9532338        |     .67021    .9989048 (exact)
 Attr. frac. pop |        .8936567        |
                 +-------------------------------------------------
                               chi2(1) =    15.51  Pr>chi2 = 0.0001
```

GFR > 140 ml/min/1.73m^2

```
. cci 22 5 11 18
                                                         Proportion
                 |   Exposed   Unexposed  |      Total     Exposed
-----------------+------------------------+------------------------
           Cases |        22           5  |         27      0.8148
        Controls |        11          18  |         29      0.3793
-----------------+------------------------+------------------------
           Total |        33          23  |         56      0.5893
                 |                        |
                 |      Point estimate    |    [95% Conf. Interval]
                 |------------------------+------------------------
      Odds ratio |             7.2        |   1.852659    30.61573 (exact)
 Attr. frac. ex. |        .8611111        |   .4602354     .967337 (exact)
 Attr. frac. pop |        .7016461        |
                 +-------------------------------------------------
                               chi2(1) =    10.96  Pr>chi2 = 0.0009
```
