# Supplementary material for: Biomarkers of sickle cell nephropathy in Senegal
Source: PLoS One. 2022 Nov 21;17(11):e0273745. doi: 10.1371/journal.pone.0273745 (PMC9678278; doi:10.1371/journal.pone.0273745)
Supplement: S2 Data — (DOCX) [file pone.0273745.s005.docx]

**List of Human Genes**

HGNC data for HBB

**Approved symbol**HBB

**Approved name**hemoglobin subunit beta

**Locus type**gene with protein product

**HGNC ID**HGNC: 4827

**Symbol status**Approved

**Previous names**hemoglobin, beta

**Alias symbols**CD113t-C, beta-globin

**Chromosomal location**11p15.4

**Gene groups :**[Hemoglobin subunits](https://www.genenames.org/data/genegroup/#!/group/940)

HGNC data for HBG2

**Approved symbol**HBG2

**Approved name**hemoglobin subunit gamma 2

**Locus type**gene with protein product

**HGNC ID**HGNC: 4832

**Symbol status** Approved

**Previous names**hemoglobin, gamma G

**Alias symbols**HBG-T1

**Chromosomal location**11p15.4

**Gene groups**[Hemoglobin subunits](https://www.genenames.org/data/genegroup/#!/group/940)
