# Supplementary material for: Biomarkers of sickle cell nephropathy in Senegal
Source: PLoS One. 2022 Nov 21;17(11):e0273745. doi: 10.1371/journal.pone.0273745 (PMC9678278; doi:10.1371/journal.pone.0273745)
Supplement: Attachment — Submitted filename: PONE-D-21-14690.pdf [file pone.0273745.s006.pdf]

PLOS ONE

BIOMARKERS OF SICKLE CELL NEPHROPATHY IN SENEGAL

--Manuscript Draft--

Manuscript Number:	PONE-D-21-14690
Article Type:	Research Article
Full Title:	BIOMARKERS OF SICKLE CELL NEPHROPATHY IN SENEGAL
Short Title:	BIOMARKERS OF SICKLE CELL NEPHROPATHY IN SENEGAL
Corresponding Author:	El Hadji Malick NDOUR, M.D.<br>Université Cheikh Anta Diop Faculté de Medecine Pharmacie d'Odonto-Stomatologie: Universite Cheikh Anta Diop Faculte de Medecine de Pharmacie et d'Odontologie<br>Dakar, SENEGAL
Keywords:	Sicile cell disease; Kidney disease; Molecular diagnostics
Abstract:	Sickle cell anemia (SCA) is caused by a single point variation in the β-globin gene (HBB): c.20A> T (p.Glu7Val), in homozygous state. SCA is characterized by sickling of red blood cells in small blood vessels which leads to a range of multiorgan complications, including kidney dysfunctions. The aim of this case-control study was to identify sickle cell nephropathy biomarkers in a group of patients living with SCA from Senegal. A total of 163 patients living with SCA and 177 ethno-linguistic matched controls were investigated. Biological phenotyping included evaluation of glycemia, glucosuria, albuminuria, proteinuria, tubular proteinuria, creatininemia, creatininuria, urine specific gravity and glomerular filtration rate. Descriptive statistics of biomarkers were performed using the $\chi 2$ – test, with the significance level set at p˂0.05. Patients with SCA had a median age of 20 years (range 4 to 57) with a sex ratio (male/female) of 1.14. The median age of the control participants was 29 years (range: 4 – 77), with 34 % being male. The following proportions of abnormal biological indices were observed in SCA patients versus (vs.) controls, as follows: hyposthenuria: 35.3 % vs. 5.2 % ($p < 0.001$) ; glomerular hyperfiltration: 47.29 % vs. 19.75 % ($p < 0.001$), chronic renal failure: 5.43 % vs. 3.82 % ($p = 0.62$)); microalbuminuria: 42.38 % vs. 5.78 % ($p < 0.001$) ; proteinuria: 39.33 % vs. 4.62 % ($p < 0.001$); tubular proteinuria: 40.97 % vs. 4.73 % ($p < 0.001$) and microglucosuria: 22.5 % vs. 5.1 % ($p < 0.001$). The present study showed a relatively high proportion of SCA nephropathy among patients living with SCA in Senegal. Microglucosuria, proteinuria, tubular proteinuria, microalbuminuria, hyposthenuria and glomerular hyperfiltration were the most prevalent biomarkers of nephropathy in this group of Senegalese patients with SCA.
Order of Authors:	El Hadji Malick NDOUR, M.D.
Additional Information:	
**Question**	**Response**
**Financial Disclosure**<br><br>Enter a financial disclosure statement that describes the sources of funding for the work included in this submission. Review the submission guidelines for detailed requirements. View published research articles from *PLOS ONE* for specific examples.<br><br>This statement is required for submission and **will appear in the published article** if the submission is accepted. Please make sure it is accurate.	The clinical chemistry experiments of the study were in part funded by The African Center of Excellence for Maternal and Child Health «Centre d'Excellence Africain pour la Santé de la Mère et de l'Enfant (CEA-SAMEF, http://ceasamef.sn, No 000099/2018/JCM/KND) », Cheikh Anta Diop University of Dakar, Senegal.<br>The molecular experiments of the study were funded by SADaCC (https://www.sickleinafrica.org) at the Division of Human Genetics, Faculty for Health Sciences, University of Cape Town, South Africa.<br>The funders had no role in study design, data collection and analysis, decision to publish, or preparation of the manuscript.

Powered by Editorial Manager® and ProduXion Manager® from Aries Systems Corporation

**Unfunded studies**<br>Enter: *The author(s) received no specific funding for this work.*<br><br>**Funded studies**<br>Enter a statement with the following details:<br>• Initials of the authors who received each award<br>• Grant numbers awarded to each author<br>• The full name of each funder<br>• URL of each funder website<br>• Did the sponsors or funders play any role in the study design, data collection and analysis, decision to publish, or preparation of the manuscript?<br>• **NO** - Include this sentence at the end of your statement: *The funders had no role in study design, data collection and analysis, decision to publish, or preparation of the manuscript.*<br>• **YES** - Specify the role(s) played.<br><br>* typeset	
**Competing Interests**<br><br>Use the instructions below to enter a competing interest statement for this submission. On behalf of all authors, disclose any competing interests that could be perceived to bias this work—acknowledging all financial support and any other relevant financial or non-financial competing interests.<br><br>This statement is required for submission and **will appear in the published article** if the submission is accepted. Please make sure it is accurate and that any funding sources listed in your Funding Information later in the submission form are also declared in your Financial Disclosure statement.<br><br>View published research articles from *PLOS ONE* for specific examples.	The authors have declared that no competing interests exist.

**NO authors have competing interests**<br><br>Enter: *The authors have declared that no competing interests exist.*<br><br>**Authors with competing interests**<br><br>Enter competing interest details beginning with this statement:<br><br>*I have read the journal's policy and the authors of this manuscript have the following competing interests: [insert competing interests here]*<br><br>* typeset	
**Ethics Statement**<br><br>Enter an ethics statement for this submission. This statement is required if the study involved:<br><br>• Human participants<br>• Human specimens or tissue<br>• Vertebrate animals or cephalopods<br>• Vertebrate embryos or tissues<br>• Field research<br><br>Write "N/A" if the submission does not require an ethics statement.<br><br>General guidance is provided below. Consult the submission guidelines for detailed instructions. **Make sure that all information entered here is included in the Methods section of the manuscript.**	The study protocol complied with the ethical guidelines of the Helsinki Declaration and was approved by the Research Ethics Committee from the Cheikh Anta Diop University of Dakar (0312/2018/CER/UCAD) and by the Faculty of Health Sciences Human Research Ethics Committee from the University of Cape Town (HREC RE: 661/2015). Participation was subject to the free and informed consent of subjects who were at least 18 years old and parents or guardians of those under 18 years.

**Format for specific study types**<br><br>**Human Subject Research (involving human participants and/or tissue)**<br>• Give the name of the institutional review board or ethics committee that approved the study<br>• Include the approval number and/or a statement indicating approval of this research<br>• Indicate the form of consent obtained (written/oral) or the reason that consent was not obtained (e.g. the data were analyzed anonymously)<br><br>**Animal Research (involving vertebrate animals, embryos or tissues)**<br>• Provide the name of the Institutional Animal Care and Use Committee (IACUC) or other relevant ethics board that reviewed the study protocol, and indicate whether they approved this research or granted a formal waiver of ethical approval<br>• Include an approval number if one was obtained<br>• If the study involved *non-human primates*, add *additional details* about animal welfare and steps taken to ameliorate suffering<br>• If anesthesia, euthanasia, or any kind of animal sacrifice is part of the study, include briefly which substances and/or methods were applied<br><br>**Field Research**<br><br>Include the following details if this study involves the collection of plant, animal, or other materials from a natural setting:<br>• Field permit number<br>• Name of the institution or relevant body that granted permission	
**Data Availability**<br><br>Authors are required to make all data underlying the findings described fully available, without restriction, and from the time of publication. PLOS allows rare exceptions to address legal and ethical concerns. See the PLOS Data Policy and FAQ for detailed information.	Yes - all data are fully available without restriction

A Data Availability Statement describing where the data can be found is required at submission. Your answers to this question constitute the Data Availability Statement and **will be published in the article**, if accepted.<br><br>**Important:** Stating 'data available on request from the author' is not sufficient. If your data are only available upon request, select 'No' for the first question and explain your exceptional situation in the text box.<br><br>Do the authors confirm that all data underlying the findings described in their manuscript are fully available without restriction?	
**Describe where the data may be found in full sentences. If you are copying our sample text, replace any instances of XXX with the appropriate details.**<br><br>• If the data are **held or will be held in a public repository**, include URLs, accession numbers or DOIs. If this information will only be available after acceptance, indicate this by ticking the box below. For example: *All XXX files are available from the XXX database (accession number(s) XXX, XXX.).*<br>• If the data are all contained **within the manuscript and/or Supporting Information files**, enter the following: *All relevant data are within the manuscript and its Supporting Information files.*<br>• If neither of these applies but you are able to provide **details of access elsewhere**, with or without limitations, please do so. For example:<br><br>*Data cannot be shared publicly because of [XXX]. Data are available from the XXX Institutional Data Access / Ethics Committee (contact via XXX) for researchers who meet the criteria for access to confidential data.*<br><br>*The data underlying the results presented in the study are available from (include the name of the third party*	All relevant data are within the manuscript and its Supporting Information files.

and contact information or URL).<br>• This text is appropriate if the data are owned by a third party and authors do not have permission to share the data.<br><br>* typeset	
Additional data availability information:	Tick here if your circumstances are not covered by the questions above and you need the journal's help to make your data available.

Powered by Editorial Manager® and ProduXion Manager® from Aries Systems Corporation

Manuscript

Click here to access/download;Manuscript;Biomarkers of sickle cell anemia. Malick Ndour, 2021. MIS.docx

Research article

BIOMARKERS OF SICKLE CELL NEPHROPATHY IN SENEGAL

El Hadji Malick Ndour[1,2*], Khuthala Mnika[3], Fatou Guèye Tall[1,2], Moussa SECK[4], Indou Dème Ly[2], Victoria Nembaware[3], Gaston Kuzamunu Mazandu[3], Hélène Ange Thérèse Sagna-Bassène[2], Rokhaya Dione[2], Aliou Abdoulaye Ndongo[5], Jean Pascal Demba Diop[6], Nènè Oumou kesso Barry[1], Moustapha Djité[1] Rokhaya Ndiaye Diallo[6], Papa Madièye Guèye[1], Saliou Diop[5], Ibrahima Diagne[7], Aynina Cissé[1], Ambroise Wonkam[3], Philomène Lopez Sall[1,2]

Affiliations:

1. Laboratoire de Biochimie Pharmaceutique, Faculté de Médecine, de Pharmacie et d'Odontologie, Université Cheikh Anta Diop, Dakar, Sénégal
2. Centre Hospitalier National d'Enfants Albert Royer, Dakar, Sénégal
3. Division of Human Genetics, Department of Pathology, Faculty of Health Sciences, University of Cape Town, Cape Town, South Africa
4. Centre National de Transfusion Sanguine, Dakar, Sénégal
5. Service de Pédiatrie de l'Hôpital Aristide Le Dantec, Dakar, Sénégal
6. Service de Génétique Humaine, Faculté de Médecine, de Pharmacie et d'Odontologie, Université Cheikh Anta Diop, Dakar, Sénégal
7. Unité de Formation et de Recherche des Sciences de la Santé, Université Gaston Berger, Saint-Louis, Sénégal

Corresponding author :

Email : elhadjimalickndour@yahoo.fr (EHMN)

Abstract

Sickle cell anemia (SCA) is caused by a single point variation in the β-globin gene (*HBB*): c.20A> T (p.Glu7Val), in homozygous state. SCA is characterized by sickling of red blood cells in small blood vessels which leads to a range of multiorgan complications, including kidney dysfunctions. The aim of this case-control study was to identify sickle cell nephropathy biomarkers in a group of patients living with SCA from Senegal. A total of 163 patients living with SCA and 177 ethno-linguistic matched controls were investigated. Biological phenotyping included evaluation of glycemia, glucosuria, albuminuria, proteinuria, tubular proteinuria, creatininemia, creatininuria, urine specific gravity and glomerular filtration rate. Descriptive statistics of biomarkers were performed using the $\chi 2$ – test, with the significance level set at $p<0.05$. Patients with SCA had a median age of 20 years (range 4 to 57) with a sex ratio (male/female) of 1.14. The median age of the control participants was 29 years (range: 4 – 77), with 34 % being male. The following proportions of abnormal biological indices were observed in SCA patients versus (vs.) controls, as follows: hyposthenuria: 35.3%vs.5.2% ($p<10^{-3}$); glomerular hyperfiltration: 47.29%vs.19.75% ($p<10^{-3}$), chronic renal failure: 5.43%vs.3.82% ($p = 6.2.10^{-1}$); microalbuminuria: 42.38 %vs.5.78% ($p<10^{-3}$); proteinuria: 39.33%vs.4.62% ($p<10^{-3}$); tubular proteinuria: 40.97%vs.4.73% ($p<10^{-3}$) and microglucosuria: 22.5%vs.5.1% ($p<10^{-3}$). The present study showed a relatively high proportion of SCA nephropathy among patients living with SCA in Senegal. Microglucosuria, proteinuria, tubular proteinuria, microalbuminuria, hyposthenuria and glomerular hyperfiltration were the most prevalent biomarkers of nephropathy in this group of Senegalese patients with SCA.

Blurb

Sickle cell anemia is a monogenic recessive autosomal inherited hemoglobinopathy which can lead to kidney disorders. This study aims at identifying relevant biomarkers for screening nephropathy in sickle cell anemia patients from Senegal. A subset of Senegalese with sickle cell anemia and ethno-linguistic matched controls both of them free from diabetes were investigated. Biological indices of renal abnormalities were evaluated and an unexpected relatively high prevalence of nephropathy among sickle cell anemia patients was revealed. Microglucosuria, proteinuria, tubular proteinuria, microalbuminuria, hyposthenuria and glomerular hyperfiltration were the most prevalent biomarkers of nephropathy in this group of Senegalese with sickle cell anemia.

Keywords: Sickle Cell Disease, Sickle Cell Nephropathy, Senegal haplotype, albuminuria, glomerular filtration rate, glucosuria, proteinuria

List of abbreviations

SCD: Sickle cell disease

NM_000518.**5**:c.20A>T: substitution of A to T at nucleotide position 20 of the complementary DNA

A: adenine

T: Thymine

NP_000509.**1**:p.Glu7Val: replacement of glutamic acid by valine at position 7 of the protein (β-globin chain)

Glu : Glutamic acid

Val : Valine

SCA: Sickle cell anemia

SNP : single nucleotide polymorphism

C : Cytosine

GFR: glomerular filtration rate

USG: urine specific gravity

ESRD: End-stage renal disease

SS: SCA patients free from diabetes

AA non-DT: control participants with no detectable SCA and diabetes

CNTS: National Blood Transfusion Center

USAD: Ambulatory Care Unit for Children and Adolescents with Sickle Cell Disease

CHEAR: Albert Royer National University Children's Hospital

CKD-EPI: Chronic Kidney Disease – EPIdemiology

UPCR: urinary protein to creatinine ratio

UACR: urinary albumin to creatinine ratio

UGCR: urinary glucose to creatinine ratio

GHF: Glomerular hyperfiltration

RFLP: restriction fragment length polymorphism

PCR: polymerase chain reaction

OR: odds ratio

USA: United State of America

mOsm/kgH_2O: urine osmolality

List of Human Genes

HGNC data for HBB

Approved symbol HBB

Approved name hemoglobin subunit beta

Locus type gene with protein product

HGNC ID HGNC: 4827

Symbol status Approved

Previous names hemoglobin, beta

Alias symbols CD113t-C, beta-globin

Chromosomal location 11p15.4

Gene groups : Hemoglobin subunits

HGNC data for HBG2

Approved symbol HBG2

Approved name hemoglobin subunit gamma 2

Locus type gene with protein product

HGNC ID HGNC: 4832

Symbol status Approved

Previous names hemoglobin, gamma G

Alias symbols HBG-T1

Chromosomal location 11p15.4

Gene groups Hemoglobin subunits

Introduction

Sickle cell disease (SCD) is an inherited hemoglobinopathy with autosomal recessive transmission caused by a single nucleotide substitution NM_000518.**5**:c.20A>T of the β-globin gene (*HBB*-rs334), located on the short arm of chromosome 11 (11p15.4) [1, 2]. The variation results in an amino-acid replacement NP_000509.**1**:p.Glu7Val of the β-globin chain of tetrameric hemoglobin ($\alpha_2\beta_2$) in adults NM_000518.**5** (HBB):c.20A>T(p.Glu7Val) [1, 2]. Sickle cell anemia (SCA) refers to the disease which results from the homozygous expression of the β^S allele ($\beta^{S/}\beta^S$ genotype) [3].

SCD is the most common monogenic disease in the world [4]. It is estimated that 305,800 children in the world, of whom 85% in sub-Saharan Africa, are born with SCD each year, and this number could reach 404,200 in 2050 [4]. Senegal is a country in sub-Saharan West Africa with a population of approximately 16,209,125, of which up to 2% are SCD patients [5, 6]. Three centers specializing in lifelong medical treatment for SCD patients have been developed, but there is no universal newborns screening yet and very few patients are exposed to hydroxycarbamide. There is no universal medical insurance coverage and care for SCD patients is thus paid for by family members in this developing country where poverty affects from 24.9% of the population living in Dakar the capital to 77.5% of the rural population of the region of Kolda [7]. Therefore, the financial burden of the necessary medical care often cannot be met and patients suffer from multiple SCD complications albeit the vast majority of the patients express the Senegal haplotype (XmnI-rs7482144) bearing the C>T single nucleotide polymorphism (SNP) at position –158 of the $^{G}\gamma$- globin gene (*HBG2*:g.-158C>T or NM_000184.**2**(HBG2):c.-211C>T) which is associated with higher fetal hemoglobin (HbF) levels known to result in a less severe clinical expression of SCD [2,3].

Patients living with SCD may exhibit multiple organ damage including renal abnormalities that may be structural and/or functional [8]. These glomerular and/or tubular renal damages are age dependent [8]. In early childhood, renal abnormalities are mainly glomerular hyperfiltration characterized by increased glomerular filtration rate (GFR), and loss of urinary concentration ability through the Henle's loop of juxtaglomerular nephrons resulting in hyposthenuria (*i.e.* a decrease in urine specific gravity (USG) are described [8]. In childhood, microalbuminuria is the most observed abnormality in SCD patients (8). In adulthood, macroalbuminuria (proteinuria) begins to develop and may be associated with renal failure (*i.e.* a decrease in GFR) [8]. End-stage renal disease (ESRD) requiring hemodialysis and / or kidney transplantation occurs in 4-18% of SCD patients [9]. The average survival time after the onset of ESRD is 4 years, and 40% of SCD patients die after 20 months of dialysis [10].

However, in Africa, particularly in Senegal, only a few studies have focused on renal manifestations of SCD, mainly on albuminuria and glomerular hyperfiltration and the available studies have been reported in patients with very low proportion of Senegal haplotype [11-14]. It is anticipated that early diagnosis in patients with one or more renal manifestations would allow early therapeutic intervention that could delay the onset of ESRD and increase the life expectancy of SCD patients [12].

Thus, the main objective of this study was to identify nephropathy biomarkers that could be used for anticipatory guidance, and affordable routine clinical assessment of Senegalese patients living with sickle cell anemia (SCA).

Results

Among 394 recruited subjects 164 (41.62%) was *HBB*-rs334 T (sickle mutation) in homozygous state, 49 (12.44%) in heterozygous state and 181 (45.94%) was *HBB*-rs334 A in homozygous state. One of the 164 β^S/β^S patients and fours of the 181 β^A/β^A one were excluded

from the study because of hyperglycemia as well as the 49 β^S/β^A subjects. A total of 163 SCA patients and 177 ethno-linguistic matched controls free from diabetes were therefore included in our series. Among the selected SCA patients 79 (63.71%) was Senegal haplotype and 45 (27.61%) were matched in age and sex with 45 of the 177 controls.Tables 1 and 2 summarize anthropometric and biochemical characteristics of the study participants. The median age of controls was 29 years [range 4 - 77] with 90% (n = 160) of this population ranging between the ages of 10 and 61. Women were more represented than men in this control group with a sex ratio of 1.95 “Table 1”. SCA patients had a median age of 20 years [range 4 - 57] and 90% (n = 147) of them were between 6 and 38 years of age. The sex ratio (F/M) was 1.14 in the SCA group “Table 2”.

Table 1: Description of anthropometric and biochemical parameters of controls

	Controls free from sickle cell anemia and diabetes (n = 177)		
	Median (min - max)	5 – 95th percentiles	Observations
Age, years	29 (4 - 77)	10 – 61	168
Sex ratio, F/M	1.95	xxx	174
BMI, kg/m²	22.38 (10.56 – 49.61)	13.52 – 34.02	162
SBP, mmHg	12 (9 -16)	10 – 14	100
DBP, mmHg	7 (5 - 9)	6 – 8	100
Hb, g/dl	13.3 (7.4 – 18.2)	10.2 – 16.2	170
Glycemia, mg/dl	84 (70 - 110)	73 - 98	170
BUN, mg/dl	8 (2.5 – 31.5)	5 - 14	162
Creatininemia, mg/dl	0.78 (0.23 – 7.36)	0.38 – 1.4	166
Creatininuria, mg/dl	227 (22 - 886)	76 - 486	174
GFR, ml/min/1.73m²	114.41 (10.85 – 200.37)	68.28 – 160.60	157
UACR, mg/g	6.52 (1.28 – 139.07)	2.62 – 32.43	173
UPCR, mg/g	44.74 (9.21 – 530.27)	15.07 - 181.48	173
UGCR, mg/g	0 (0 - 52)	0 - 20	176
USG	1.020 (1.004 – 1.035)	1.010 – 1.028	154

SBP, mmHg (x 0.134 kPa). DBP, mmHg (x 0.134 kPa). Hb, g/dl (x 0.6206 mmol/l). Glycemia, mg/dl (x 0.0555 mmol/l). BUN, mg/dl (x 0.357 mmol/l). Creatininemia, mg/dl (x 88.4 µmol/l). Creatininuria, mg/dl (x 88.4 µmol/l). UACR, mg/g (x 0.113 mg/mmol). UPCR, mg/g (x 0.113 mg/mmol). UGCR, mg/g (x 0.625 µmol/mmol).

n: ~~effectif~~. Min: minimum. Max: maximum. BMI: body mass index. SBP: systolic blood pressure. DBP: diastolic blood pressure. Hb: hemoglobin. BUN: blood urea nitrogen. GFR: glomerular filtration rate was determined using Schwartz formula in children and adolescents and CKD-EPI formula in adults. UPCR: urinary protein /creatinine ratio. UACR: urinary albumin/creatinine ratio. UGCR: urinary glucose/creatinine ratio. USG: urine specific gravity.

Table 2: Description of anthropometric and biochemical parameters of sickle cell anemia patients

	Sickle cell anemia patients free from diabetes (n = 163)		
	Median (min - max)	5 – 95th percentiles	Observations
Age, years	20 (4 - 57)	6 - 38	144
Sex ratio, F/M	1.14	xxx	156
BMI, kg/m²	18 (11.24 – 33.71)	13.15 – 26.04	128
SBP, mmHg	11 (8 - 14)	9 - 14	101
DBP, mmHg	7 (4 - 11)	5 - 8	101
Hb, g/dl	8.4 (5.1 – 11.6)	6.6 - 11	148
Glycemia, mg/dl	85 (60 - 108)	71 - 101	152
BUN, mg/dl	6.5 (2.5 – 54.5)	4 – 10.5	143
Creatininemia, mg/dl	0.57 (0.13 – 1.58)	0.27 – 0.99	151
Creatininuria, mg/dl	88 (16 - 679)	37 - 233	152
GFR, ml/min/1.73m²	136.96 (38.31 – 407.84)	58.51 -196.80	129
UACR, mg/g	25.67 (2.64 – 328.65)	6.86 – 122.63	151
UPCR, mg/g	156.67 (17.24 – 2957.84)	29.41 – 1388.76	150
UGCR, mg/g	5 (0 - 7370)	0 - 193	151
USG	1.012 (1.001 – 1.025)	1.007 – 1.020	153

SBP, mmHg (x 0.134 kPa). DBP, mmHg (x 0.134 kPa). Hb, g/dl (x 0.6206 mmol/l). Glycemia, mg/dl (x 0.0555 mmol/l). BUN, mg/dl (x 0.357 mmol/l). Creatininemia, mg/dl (x 88.4 µmol/l). Creatininuria, mg/dl (x 88.4 µmol/l). UACR, mg/g (x 0.113 mg/mmol). UPCR, mg/g (x 0.113 mg/mmol). RGCU, mg/g (x 0.625 µmol/mmol).

n: effectif. Min: minimum. Max: maximum. BMI: body mass index. SBP: systolic blood pressure. DBP: diastolic blood pressure. Hb: hemoglobin. BUN: blood urea nitrogen. GFR: glomerular filtration rate was determined using Schwartz formula in children and adolescents and CKD-EPI formula in adults. UPCR: urinary protein /creatinine ratio. UACR: urinary albumin/creatinine ratio. UGCR: urinary glucose/creatinine ratio. USG: urine specific gravity.

The median creatininemia observed in 166 controls was 0.78 mg/dl (68.95 µmol/l) [range 0.23 – 7.36 (20.33 - 650.62)] with 90% (n = 150) of this group having creatininemia between 0.38 (33.59 µmol/l) and 1.4 mg/dl (123.76 µmol/l) "Table 1". The reference intervals of creatininemia obtained from the controls of our series were therefore 0.78 mg/dl (68.95 µmol/l) [range 0.38-1.4 (33.59 - 123.76)] "Table 1" [15]. The mean creatininemia level was significantly reduced in SCA compared to controls [0.59±0.23 (52.15±20.33) versus 0.86±0.60 (76.02±53.04) mg/dl (µmol/l); $p < 10^{-4}$] "Table 3". The median glomerular filtration rate (GFR) was 136.96 ml/min /1.73m^2 [range 58.51- 196.80] for SCA patients and 114.41 ml/min/1.73m^2 [range 68.28 – 160.60] for controls "Tables 1, 2".The median urinary specific gravity (USG) was 1.012 [range: 1.007 - 1.020] and USG was ≤ 1.015 in 90.2% (n = 138) of SCA patients "Tables 2, 4"**.** In controls, the 5th percentile of USG was 1.010 "Table 1". Hyposthenuria could therefore be defined by an USG ≤ 1.010 [15]. The 95th percentile for glucosuria, albuminuria, and proteinuria were respectively 20 mg/g (12.5 µmol/mmol), 32.43 mg/g (3.66 mg/mmol) and 181.48 mg/g (20.51 mg/mmol) in the control group "Table 1". Microglucosuria could therefore be defined by an UGCR ≥ 20 mg/g (12.5 µmol/mmol) but could remain low and undetectable by urine test strips.

Table 3: Comparison of means of anthropometric and biochemical parameters between sickle cell anemia patients and unmatched controls and then age- and sex-matched controls.

	SS (n = 163)	AA non-DT (n = 177)		SS (n = 45)	AA non DT (n = 45)	
	Mean±SD or % (n)	Mean±SD or % (n)	p-value	Mean±SD or % (n)	Mean±SD or % (n)	p-value
Age, years	20.4±10.2	32.1±15.3	$< 10^{-4}$	21.4±10.4	21.4±10.4	1
Sex, female	53.21% (83)	66.09%(115)		64.44%(29)	64.44%(29)	
Sex, male	46.79% (73)	33.91% (59)	$1.7.10^{-2}$	35.56%(16)	35.56%(16)	1
BMI, kg/m²	19.1± 6.8	22.9±6.8	$< 10^{-4}$	19.6±7.5	21,5±6.8	$7.9.10^{-2}$
SBP, mmHg	11.2±1.30	11.7±1.35	$4.1.10^{-3}$	11.2±1.15	11.8±1.01	$7.6.10^{-2}$
DBP, mmHg	6.9±1.12	7.25±0.8	$8.5.10^{-3}$	7.25±1.06	7.35±0.79	$3.96.10^{-1}$
Hb, g/dl	8.57±1.47	13.38±1.8	$< 10^{-4}$	8.69±1.43	13.05±1.87	$< 10^{-4}$
Glycemia, mg/dl	85±9	86±8	$7.1.10^{-1}$	85±7	86±10	$4.47.10^{-1}$
BUN, mg/dl	7±4.5	9±3.5	$< 10^{-4}$	6.5±2	8.5±2.5	3.10^{-4}
Creatininemia, mg/dl	0.59±0.23	0.86±0.60	$< 10^{-4}$	0.58±0.21	0.75±0.32	$9.5.10^{-3}$

SBP, mmHg (x 0.134 kPa). DBP, mmHg (x 0.134 kPa). Hb, g/dl (x 0.6206 mmol/l). Glycemia, mg/dl (x 0.0555 mmol/l). BUN, mg/dl (x 0.357 mmol/l). Creatininemia, mg/dl (x 88.4 µmol/l).

SS: sickle cell anemia patients free from diabetes. AA non-DT: controls free from sickle cell anemia and diabetes. n: effectif ; BMI: body mass index. SBP: systolic blood pressure. DBP: diastolic blood pressure. Hb: hemoglobin. BUN : blood urea nitrogen.

In the SCA group, the prevalence was 35.3% for hyposthenuria, 47.29% for glomerular hyperfiltration, 5.43% for chronic renal failure, 42.38% for microalbuminuria, 39.33% for proteinuria, 0% for glomerular hyperproteinuria, 40.97% for tubular hyperproteinuria and 22.5% for microglucosuria “Table 4”. In the control group, the prevalence was 5.2% for hyposthenuria, 19.75% for glomerular hyperfiltration, 3.82% for chronic renal failure, 5.78% for microalbuminuria, 4.62% for proteinuria, and 0% for glomerular hyperproteinuria, 4.73%

for tubular hyperproteinuria and 5.1% for microglucosuria “Table 4”. Comparison of prevalence between SCA patients and unmatched controls showed that SCA was associated with renal injury biomarkers ($p < 0.05$) except for CRF ($p = 6.2 \times 10^{-1}$), and glomerular hyperproteinuria “Table 4”. This association was strong and SCA appeared as a risk factor of renal insult biomarkers disruption if the odds ratio (OR) was taken into account “Table 4”. The association between SCA and these renal disorders were confirmed by comparing the prevalence of these nephropathy biomarkers between SCA patients and controls matched in age and sex “Table 5”.

Table 4: Comparison of disturbances of nephropathy biomarkers between sickle cell anemia patients and controls

	SS (n = 163)	AA non-DT (n = 177)		
	% (n)	% (n)	p-value	Odds ratio
USG ≤ 1.010	35.3%(54)	5.2%(8)		
USG > 1.010	64.7%(99)	94.8%(146)	$< 10^{-3}$	9.95 [4.43-25.12]
GFR > 140 ml/min/1.73 m²	47.29%(61)	19.75%(31)	$< 10^{-3}$	3.64 [2 – 6.64]
GFR < 60 ml/min/1.73 m²	5.43% (7)	3.82%(6)	$6.2.10^{-1}$	
60 ≤ GFR < 90 ml/min/1.73 m²	11.63%(15)	22.29%(35)		
90 ≤ GFR ≤ 140 ml/min/1.73 m²	35.66%(46)	54.14%(85)		
UACR ≥ 30 mg/g	42.38%(64)	5.78%(10)		
UACR < 30 mg/g	57.62%(87)	94.22%(163)	$< 10^{-3}$	11.99 [5.71-27.33]
UPCR > 200 mg/g	39.33%(59)	4.62%(8)		
UPCR ≤ 200 mg/g	60.67%(91)	95.38%(165)	$< 10^{-3}$	13.37 [5.97-33.59]
UPCR > 200 mg/g with $\frac{UACR}{UPCR} \geq 59\%$	0	0		
UPCR ≤ 200 mg/g with $\frac{UACR}{UPCR} \geq 59\%$	6	4	NA	NA
UPCR > 200 mg/g with $\frac{UACR}{UPCR} < 59\%$	40.97%(59)	4.73%(8)		
UPCR ≤ 200 mg/g with $\frac{UACR}{UPCR} < 59\%$	59.03%(85)	95.27%(161)	$< 10^{-3}$	13.97 [6.22-35.15]
UGCR ≥ 20 mg/g	22.5%(34)	5.1%(9)		
UGCR < 20 mg/g	77.5%(117)	94.9%(167)	$< 10^{-3}$	5.39 [2.41-13.21]

UACR, mg/g (x 0.113 mg/mmol). UPCR, mg/g (x 0.113 mg/mmol). UGCR, mg/g (x 0.625 µmol/mmol).

SS : sickle cell anemia patients free from diabetes. AA non-DT : controls free from sickle cell anemia and diabetes. n : effectif. USG : urine specific gravity. GFR : glomerular filtration rate was determined using Schwartz formula in children and adolescents and CKD-EPI formula in adults. UPCR : urinary protein /creatinine ratio. UACR : urinary albumin/creatinine ratio. RACU/RPCU : urinary albumine/total protein ratio. UGCR : urinary glucose/creatinine ratio. NA : not applicable.

Table 5: Comparison of disturbances of nephropathy biomarkers between sickle cell anemia patients and age- and sex-matched controls

	SS (n = 45)	AA non-DT (n = 45)		
	% (n)	% (n)	p-value	Odds ratio
USG ≤ 1.010	35.71%(15)	2.44 %(1)		
USG > 1.010	64.29%(27)	97.56%(40)	$< 10^{-3}$	22 [3 - 958]
GFR > 140 ml/min/1.73 m²	45.24%(19)	21.43%(9)	$4.9.10^{-2}$	2.73 [0.89 – 8.61]
GFR < 60 ml/min/1.73 m²	2.38%(1)	2.38%(1)	$8.6.10^{-1}$	
60 ≤ GFR < 90 ml/min/1.73 m²	11.90%(5)	23.81%(10)	$5.6.10^{-1}$	
90 ≤ GFR ≤ 140 ml/min/1.73 m²	40.48%(17)	52.38%(22)		
UACR ≥ 30 mg/g	47.73%(21)	8.89%(4)		
UACR < 30 mg/g	52.27%(23)	91.11%(41)	$< 10^{-3}$	9.36 [2.64 – 41.02]
UPCR > 200 mg/g	37.21%(16)	2.22%(1)		
UPCR ≤ 200 mg/g	62.79%(27)	97.78%(44)	$<10^{-3}$	26.07 [3.5 -1117.4]
UPCR > 200 mg/g with $\frac{UACR}{UPCR} \geq 59\%$	0	0		
UPCR ≤ 200 mg/g with $\frac{UPCR}{UPCR} \geq 59\%$	2	0	NA	NA
UPCR > 200 mg/g with $\frac{UACR}{UPCR} < 59\%$	39.02%(16)	2.22%(1)		
UPCR ≤ 200 mg/g with $\frac{UACR}{UPCR} < 59\%$	60.98%(25)	97.78%(44)	$< 10^{-3}$	28.2 [3.8-1206.7]
UGCR ≥ 20 mg/g	25%(11)	6.67%(3)		
UGCR < 20 mg/g	75%(33)	93.33%(42)	$1.8.10^{-2}$	4.67 [1.09-27.69]

UACR, mg/g (x 0.113 mg/mmol). UPCR, mg/g (x 0.113 mg/mmol). UGCR, mg/g (x 0.625 μmol/mmol).

SS: sickle cell anemia patients free from diabetes. AA non-DT: controls free from sickle cell anemia and diabetes. n: ~~effectif~~. USG: urine specific gravity. GFR: glomerular filtration rate was determined using Schwartz formula in children and adolescents and CKD-EPI formula in adults. UPCR: urinary protein /creatinine ratio. UACR: urinary albumin/creatinine ratio. RACU/RPCU: urinary albumine/total protein ratio. UGCR: urinary glucose/creatinine ratio. NA: not applicable.

Discussion

To our knowledge, this is the first study to investigate renal abnormalities in a group of patients living with SCA from Senegal, which revealed a relatively high proportion of patients with such a broad spectrum of abnormal biological indices of renal disorders. This was to some extend unexpected as most patients have the relatively favorable Senegal haplotype. This research will contribute to fill the gap of investigation of renal insult in African cohort and emphasize the need to improve prevention and care for all SCD patients in Africa irrespective of their genetic and regional background.

This study confirms that loss of the ability to concentrate urine appears to be the most common renal functional impairment in SCA, in line with a comparable prevalence of 94.8% that was reported among Americans living with SCD [16]. Hyposthenuria was defined, in our study, as an USG $\leq$ 1.010 which is equivalent to 400 milliosmoles according to the equation $mOsm/kgH_2O$ = (USG -1,000) x 40,000 where $mOsm/kgH_2O$ represents the urine osmolality and USG the urine specific gravity [17]. Hence, 400 milliosmoles would precisely represent the maximum concentration ability of the cortical nephrons and the minimum concentration ability of the juxta-glomerular nephrons associated with vasa recta [18]. Concentrating the urine beyond 400 milliosmoles would therefore require the intervention of the juxta-glomerular nephrons which, in association with the vasa recta, ensure the mechanism of the counter-current multiplication which concentrates the urine beyond 400 milliosmoles. In patients with SCA, the hypoxic, hyperosmolar and acidic medullary environment promotes sickling of the red blood cells [18]. Recurrent cycles of ischemia-reperfusion eventually destroy the vasa recta and the juxta-glomerular nephrons then lose their ability to concentrate urine, which becomes hyposthenuric [18]. This could explain why 35.3% of SCA patients had hyposthenuria compared with only 5.2% of controls with a significant difference ($p < 10^{-3}$) and an OR = 9.95 (95% CI: 4.43-25.12) "Table 4".

GFR is the best renal biomarker that provides an overall assessment of kidney function. There is no consensus yet on the formula to be used to determine GFR in SCA patients. Similar to other authors, we used the Schwartz's formula in children and the CKD-EPI in adults to determine the GFR, and found a median value of 136.96 ml/min/1.73m^2, that was comparable to those described among youngest Senegalese patients with SCD (130 ml/min/1.73m^2), Cameroonian SCD patients (135.1 ml/min/1.73m^2), Ghanaian SCD patients (136.09 ml/min/1.73m^2) and Jamaican SCD patients (137 ml/min /1.73m^2) “Table 2” [12, 14, 19, 20, 21]. In the absence of a consensual threshold, glomerular hyperfiltration (GHF) was defined, in our study, by a GFR > 140 ml/min/1.73m^2 without distinction of age or sex, like some authors [20, 22, 23**]**. The prevalence of GHF in SCA patients was 47.29% comparable to that found among American with SCD in Tennessee (47%) and among Cameroonian living with SCD (49.5%) **“**Table 5” [14, 24]**.**

A theory to explain the occurrence of GHF in SCA has already been formulated [8, 25]. Nevertheless, we have tried to propose a new one as both hyposthenuria and GHF would be statistically attributable to SCA. In healthy subjects, a decrease in GFR following a drop in blood pressure (BP) leads to a reduction in tubular osmolality [26]. In response to the hypo-osmolality detected by its osmoreceptors, the macula densa triggers tubulo-glomerular feedback mechanism and initiates hormonal regulation to increase GFR [26]. In SCA patients, the macula densa triggers these mechanisms to increase GFR assumed to be decreased. This results in GHF since the hypo-osmolality in SCA is not from a decrease in GFR but a loss of the concentration ability of the juxta-glomerular nephrons.

Over time, GHF could lead to glomerulosclerosis and possibly to kidney failure [27]. The prevalence of chronic renal failure found in this study (5.43%) was comparable to what previously reported among Americans with SCD (4%), similarly in SCD patients living in Brazil (5.1%) and India (5.68) “Table 5” [20, 24, 28, 29].

With SCA, the kidney undergoes structural lesions that may manifest as proteinuria, albuminuria and eventually microglucosuria. The 95th percentile of UACR in controls (32.43 mg/g [3.66 mg/mmol]) is close to the conventional lower limit that defines microalbuminuria (30 mg/g [3.39 mg/mmol]) “Table 1”. Considering the conventional value, the prevalence of microalbuminuria was 42.38% among SCA patients in this study and was comparable to those found by a multicenter study in an African pediatric patients with SCA (36%) and with a multicenter study in two adult American cohorts (43.5%, 42%) and among a Nigerian group of patients with SCA (42.7%) “Table 4” [11, 27, 30, 31]. Macroalbuminuria was observed in our series only in a 10-year-old girl. While macroalbuminuria always indicates glomerulopathy, the clinical significance, specifically the glomerular and/or tubular origin of microalbuminuria, is not yet clearly established in patients with SCA [23, 27].

In healthy subjects, proteinuria can reach 180 mg/g (20.34 mg/mmol) [32]. In our study, the 95th percentile of the UPCR in controls (181.48 mg/g [20.51 mg/mmol]) is close to the upper limit of normal proteinuria “Table 1” [32]. The prevalence of proteinuria (UPCR > 200 mg/g [22.6 mg/mmol]) found among Senegalese SCA patients (39.33%) was comparable to data from Ghana (40.8%), Saudi Arabia (41%) and the USA (41%) “Table 4” [19, 33, 34, 35].

The urinary albumin / total protein ratio (UACR/UPCR) was used to investigate whether the proteinuria could be of glomerular or tubular origin. Pathological proteinuria (UPCR > 200 mg/g [22.6 mg/mmol]) of glomerular origin would consist of more than 59% albumin (UACR/UPCR ≥ 59%) otherwise it would be of tubular origin [33, 36]. Proteinuria was, as expected, physiological (UPCR ≤ 200 mg/g [22.6 mg/mmol]) and of tubular origin (UACR/UPCR < 59%) in 95.27% of controls “Table 4”. No pathological proteinuria (UPCR > 200 mg/g [22.6 mg/mmol]) was of glomerular origin (UACR/UPCR ≥ 59%) in either SCA patients or controls “Table 4”. In contrast, pathological proteinuria (UPCR > 200 mg/g [22.6 mg/mmol]) of tubular origin (UACR/UPCR < 59%) or tubular proteinuria was recorded in

40.97% of SCA patients and in 4.73% of controls with a significant difference ($p < 10^{-3}$) and an *OR* = 13.97 (95%CI: 6.22-35.15) “Table 4”. This would mean that proteinuria in SCA patients results more from tubular lesions than from glomerular damage. If this hypothesis was true, the tubular reabsorption of filtered glucose could be impaired and microglucosuria could be found in SCA patients “Table 4”. Thus, the prevalence of microglucosuria 22.5% found in this study provide evidence that proteinuria or even microalbuminuria could be caused by a decrease in tubular reabsorption of filtered proteins due to competition between hemoglobin and filtered proteins on the receptors of the proximal tubule or due to heme-induced proximal tubule lesions [37, 38]. Proximal tubule cells damaged in this way may no longer participate in the reabsorption of filtered proteins, albumin in particular and glucose, promoting the occurrence of tubular proteinuria, microalbuminuria and microglucosuria.

However, our study presented some limitations. First, it was impossible for us to assess whether the biomarkers were transiently or permanently disturbed since this was a case-control study. Second, odds ratios, which were very high in some cases, would reflect the presence of confounding factors that only a multivariate analysis could remove. Third, the serologic assays of *hepatitis B virus*, *Streptococcus pneumoniae or Schistosoma haematobium* had not been carried out though these pathogens may cause the kidney disorders in some controls or patients with SCA

In conclusion, this study showed a relatively high proportion of SCA nephropathies among patients living with SCA in Senegal. The study emphasizes that hyposthenuria, glomerular hyperfiltration, microalbuminuria, tubular proteinuria and microglucosuria could be relevant biomarkers of sickle cell nephropathy. Our study revealed a new biomarker, microglucosuria, which could be used as well as the urinary albumin/total protein ratio in association with proteinuria to diagnose renal tubular lesions in sickle cell anemia patients. The study to identify anthropometric, clinico-biological, genetic and even environmental risk factors that predispose

these biomarkers to disturbances will be necessary to be able to identify at-risk patients and allow early detection and therapeutic management of sickle cell nephropathy.

Materials and Methods

The study protocol complied with the ethical guidelines of the Helsinki Declaration and was approved by the Research Ethics Committee from the Cheikh Anta Diop University of Dakar (0312/2018/CER/UCAD) and by the Faculty of Health Sciences Human Research Ethics Committee from the University of Cape Town (HREC RE: 661/2015). Participation was subject to the free and informed consent of subjects who were at least 18 years old and parents or guardians of those under 18 years.

This was a case-control study that included SCA patients free from diabetes (SS) and controls with no detectable SCA and diabetes (AA non-DT).

Patients with SCA were recruited in Dakar (Senegal) at the National Blood Transfusion Center «Centre National de Transfusion Sanguine (CNTS)», the reference care center for adults with SCA; and the Ambulatory Care Unit for Children and Adolescents with Sickle Cell Disease «Unité de Soins Ambulatoires des enfants et adolescents atteints de Drépanocytose (USAD)» located at the Albert Royer National University Children's Hospital «Centre Hospitalier National d'Enfants Albert Royer (CHEAR) », the largest care unit for children and adolescents with SCA in Senegal. The control participants were recruited during two campaigns of free medical consultations organized in two suburbs of Dakar. Patients with SCA were included in the study if they were already enrolled in the sickle cell adult or infant cohort, at least 4 years of age, at a routine fasting visit, and in steady state health. The exclusion criteria included those in a pain crisis and/or with diabetes. Samples from the control participants were collected when they were apparently healthy and at least 4 years old. Control participants were excluded from

the study when their hemoglobin solubility test was positive and their *HBB* genotype was β^S/β^A and/or their fasting blood sugar ≥ 1.26 g/l.

The assessment of biological indices was conducted at the Clinical Chemistry Laboratory of Albert Royer National University Children's Hospital of Dakar (CHEAR). The quantitative determination of hemoglobin with sodium lauryl sulfate, a cyanide-free reagent, was performed using the Sysmex XT-4000i (Sysmex Corporation, Kobe, Japan). Using a Mindray-BS-380 clinical biochemistry analyzer (Mindray, Créteil, France) and Biosystems reagents (Biosystems reagents & instruments, Barcelone, Espagne), the following parameters were quantitatively determined by spectrocolorimetry: glycemia and glucosuria using glucose oxidase / peroxidase system, blood urea nitrogen (BUN) using urease / Berthelot reagent system, creatininemia and creatininuria using creatininase / creatinase / sarcosine oxidase / peroxidase enzymatic system with standardization to isotope dilution mass spectrometry, proteinuria using pyrogallol red, albuminuria using specific anti-human albumin antibodies by immunoturbidimetry. Glucose was also tested in urine using glucose oxidase / peroxidase activity of the urine test strips (nal von minden GmbH, Regensburg, Allemagne). Urine specific gravity was measured using an Atago-SPR-T2 refractometer (Atago, Saitama, Japon). GFR was computed using Schwartz's formula in children and adolescents, and Chronic Kidney Disease - EPIdemiology (CKD-EPI) equation in adults [39, 40].

Proteinuria and albuminuria were normalized with creatininuria and expressed as a ratio. Thus, proteinuria was expressed as a urinary protein to creatinine ratio (UPCR) and albuminuria as a urinary albumin to creatinine ratio (UACR). All two ratios were expressed as mg of protein or albumin per g of urine creatinine (mg/g). UPCR was defined as pathological proteinuria (UPCR > 200 mg/g [22.6 mg/mmol]) or physiological proteinuria (UPCR ≤ 200 mg/g [22.6 mg/mmol]). The urinary albumin / total protein ratio (UACR/UPCR) indicated the origin of proteinuria which was qualified as glomerular (UACR/UPCR ≥ 59%) or tubular (UACR/UPCR < 59%).

Thus, for example, normal glomerular proteinuria was defined as UPCR ≤ 200 mg/g (22.6 mg/mmol) with UACR / UPCR ≥ 59% while tubular pathologic proteinuria was defined as UPCR > 200 mg/g (22,6 mg/mmol) with UACR/UPCR < 59%. UACR was defined as normoalbuminuria (UACR < 30 mg/g [3.39 mg/mmol]), microalbuminuria (30 mg/g [3.39 mg/mmol] ≤ UACR < 300 mg/g [33.9 mg/mmol]) or macroalbuminuria (UACR ≥ 300 mg/g [33.9 mg/mmol]). Microglucosuria was defined as glucosuria which is not the consequence of hyperglycemia and which might not be detectable by urine test strips that generally do not detect glucosuria below 50 mg/dl (2.775 mmol/l) but which is quantifiable by the glucose oxidase peroxidase method which can determine glucosuria 200 times lower (0.23 mg/dl [0.013 mmol/l]) according to the manufacturers of the reagents used in our study. Glucosuria was normalized by determining the ratio of glucosuria (mg/dl) to creatininuria (g/dl), abbreviated UGCR, expressed in mg/g (x 0.625 μmol/mmol). Glucosuria greater than or equal to the 95th percentile of the UGCR in the control group was considered to be microglucosuria "Table 1". Hyposthenuria qualified an USG ≤ 5th percentile of the USG observed in the control group. Glomerular hyperfiltration (GHF) was defined by GFR > 140 ml/min/$1.73m^2$ and chronic renal failure by GFR < 60 ml/min/$1.73m^2$.

DNA was extracted from peripheral blood at the Clinical Chemistry Laboratory of Albert Royer National University Children's Hospital of Dakar (CHEAR) using Puregene Blood Kit (Qiagen, Hilden, Germany). Molecular confirmation of SCA was performed at the Division of Human Genetics, Faculty for Health Sciences, University of Cape Town using restriction fragment length polymorphism (RFLP) with the same materials and protocols previously described [14]. Molecular analysis to determine the presence of the sickle mutation was carried out by polymerase chain reaction (PCR) to amplify a 770 bp segment of *HBB*, followed by DdeI restriction analysis of the PCR product [14]. Genotyping for the *XmnI*-rs7482144 was

performed using the iPLEX Gold Sequenom Mass Genotyping Array (Inqaba Biotec, Pretoria, South Africa).

Descriptive statistics was used for anthropometric and biological variables (median, minimum, maximum, 5th and 95th percentiles), for both cases and controls. In addition, the Wilcoxon-Mann-Whitney test was used to compare the means, for quantitative variables, between cases and unmatched controls, and between cases and controls matched on age and sex. Relevant quantitative parameters of nephropathy were transformed into categorical variables. The comparison of the prevalence of biomarker disturbances was carried out using the $\chi 2$ test between unmatched cases and controls and then between cases and controls matched on age and sex. When an association was statistically established, the odds ratio (OR) was then calculated. The significance level for the tests was set at $p < 0.05$. Statistical analysis was carried out using STATA version 14.0.370 for Windows TM (Stata Corp Inc., College Station, Texas, USA).

References

1. Sundd P, Gladwin MT, Novelli EM. Pathophysiology of Sickle Cell Disease. Annu Rev Pathol 2019;14:263-92.

2. National Center of Biotechnology Information. NCBI ClinVar

   www.ncbi.nlm.nih.gov/clinvar/variation/15333/ (Accessed March 2021)

. www.ncbi.nlm.nih.gov/clinvar/variation/14984/ (Accessed March 2021)

3. Gueye Tall F, Martin C, Ndour EHM, Ly ID, Renoux C, Chillotti L, et al. Genetic Background of the Sickle Cell Disease Pediatric Population of Dakar, Senegal, and Characterization of a Novel Frameshift β-Thalassemia Mutation [HBB: c.265_266del; p.Leu89Glufs*2]. Hemoglobin 2017;41:89-95.

4. Piel FB, Patil AP, Howes RE, Nyangiri OA, Gething PW, Dewi M, et al. Global epidemiology of sickle haemoglobin in neonates: a contemporary geostatistical model-based map and population estimates. Lancet 2013;381:142–51.

5. Agence Nationale de la Statistique et de la Démographie (ANSD) de la République du Sénégal: La population du Sénégal en 2019, 2020.

6. World Health Organisation (WHO), Regional Committee for Africa Sixtieth Session Malabo, Equatorial Guinea: Sickle-Cell Disease: a strategy for the WHO African Region, 2011.

7. Banque Mondiale, Agence Nationale de la Statistique et de la Démographie (ANSD) de la République du Sénégal: Sénégal : cartes de pauvreté, édition 2011, 2016.

8. Nath KA, Hebbel R P. Sickle cell disease: renal manifestations and mechanisms. Nat Rev Nephrol 2015;11:161–71.

9. Powars DR, Elliott-Mills DD, Chan L, Niland J, Hiti AL, Opas LM, Johnson C. Chronic renal failure in sickle cell disease: risk factors, clinical course, and mortality. Ann Intern Med 1991;115:614–620.

10. Powars DR, Chan LS, Hiti A, Ramicone E, Johnson C. Outcome of sickle cell anemia: a 4-decade observational study of 1056 patients. Med Baltim 2005;84:363–376.

11. Ranque B, Thiam MM, Diallo DA, Diop S, Diagne I, Sanogo I, et al. Sickle cell disease glomerulopathy in five subsaharian african countries: results of the Cadre Study. Blood 2013;122: 779.

12. Ranque B, Menet A, Diop IB, Thiam MM, Diallo D, Diop S, et al. Early renal damage in patients with sickle cell disease in sub-Saharan Africa: a multinational, prospective, cross-sectional study. Lancet Haematol 2014;1:e64–73.

13. Kaze FF, Kengne AP, Atanga LC, Monny Lobe M, Menanga AP, Halle MP, et al. Kidney function, urinalysis abnormalities and correlates in equatorial Africans with sickle cell disease. Clin Kidney J 2013;6:15-20.

14. Geard A, Pule GD, Chemegni BC, Bitoungui VJN, Kengne AP, Chimusa ER, Wonkam A. Clinical and genetic predictors of renal dysfunctions in sickle cell anaemia in Cameroon. Br J Haematol 2017;178:629-39.

15. Ozarda Y, Higgins V, Adeli K. Verification of reference intervals in routine clinical laboratories: practical challenges and recommendations. Clin Chem Lab Med 2019;57:30–37.

16. Sundaram N, Bennett M, Wilhelm J, Kim MO, Atweh G, Devarajan P, Malik P. Biomarkers for early detection of sickle nephropathy. Am J Hematol 2011;86:559-66.

17. Chadha V, Garg U, Alon U S. Measurement of urinary concentration: a critical appraisal of methodologies. Pediatr Nephrol 2001;16:374-82.

18. Statius van Eps LW, Pinedo-Veels C, de Vries GH, de Koning J. Nature of concentrating defect in sickle-cell nephropathy. Microradioangiographic studies. Lancet 1970;1:450-2.

19. Ephraim RK, Osakunor DN, Cudjoe O, Oduro EA, Asante-Asamani L, Mitchell J, et al. Chronic kidney disease is common in sickle cell disease: a cross-sectional study in the Tema Metropolis, Ghana. BMC Nephrol 2015;16:75.

20. Lakkakula BVKS, Verma HK, Choubey M, Patra S, Khodiar PK, Patra PK. Assessment of renal function in Indian patients with sickle cell diseuse. Saudi J Kidney Dis Transpl 2017;28:524-31.

21. Thompson J, Reid M, Hambleton I, Serjant GR. Albuminuria and renal function in homozygous sickle call disease: Observations from a cohort study. Arch Intern Med 2007;167:701–8.

22. Aloni MN, Ngiyulu RM, Ekulu PM, Mbutiwi FI, Makulo JR, Gini-Ehungu JL, et al. Glomerular hyperfiltration is strongly correlated with age in Congolese children with sickle cell anaemia. Acta Paediatr 2017;106:819-24.

23. Zahr RS, Yee ME, Weaver J, Twombley K, Matar RB, Aviles D, et al. Kidney biopsy findings in children with sickle cell disease: a Midwest Pediatric Nephrology Consortium study. Pediatr Nephrol 2019;34:1435-45.

24. Gosmanova EO, Zaidi S, Wan JY, Adams-Graves PE. Prevalence and progression of chronic kidney disease in adult patients with sickle cell disease. J Investig Med 2014;62:804–7.

25. De Jong PE, Statius Van Eps LW. Sickle cell nephropathy: new insights into its pathophysiology. Kidney Int 1985:711–17.

26. Blantz RC, Deng A, Miracle CM, Thomson SC. Regulation of kidney function and metabolism: a question of supply and demand. Trans Am Clin Climatol Assoc 2007;118:23-43.

27. Guasch A, Navarrete J, Nass K, Zayas CF. Glomerular involvement in adults with sickle cell hemoglobinopathies: prevalence and clinical correlates of progressive renal failure. J Am Soc Nephrol 2006;17:2228–35.

28. Kabir OO, Allegretti AS, Zhao SH, Achebe MM, Eneanya ND, Thadhani RI, et al. Kidney function decline among black patients with sickle cell trait and sickle cell disease: an observational cohort study. J Am Soc Nephrol 2020;3:393-404.

29. Silva GBJ, Libório AB, Vieira AP, Bem AX, Lopes ASF, Figueiredo ACF, et al. Evaluation of renal function in sickle cell disease patients in Brazil. Braz J Med Biol Res 2012;45:652-5.

30. Drawz P, Ayyappan S, Nouraie M, Saraf S, Gordeuk V, Hostetter T, et al. Kidney disease among patients with sickle cell disease, hemoglobin SS and SC. Clin J Am Soc Nephrol 2016;11:207–15.

31. Solarin AU, Njokanma FO, Kehinde O. Prevalence and clinical correlates of microalbuminuria among children with sickle cell anaemia attending Lagos State University Teaching Hospital, Ikeja. Afr J Paediatr Nephrol 2014;1:37-45.

32. Bökenkamp A. Proteinuria—take a closer look! Pediatr Nephrol 2020;35:533–41.

33. Viteri B, Reid-Adam J. Hematuria and proteinuria in children. Pediatr Rev 2018;39:573–87.

34. Aleem A. Renal abnormalities in patients with sickle cell disease: a single center report from Saudi Arabia. Saudi J Kidney Dis Transpl 2008;19:194–99.

35. Marsenic O, Couloures KG, Wiley JM. Proteinuria in children with sickle cell disease. Nephrol Dial Transplant 2008;23:715–20.

36. Ohisa N, Yoshida K, Matsuki R, Suzuki H, Miura H, Ohisa Y, et al. A comparison of urinary albumin-total protein ratio to phase-contrast microscopic examination of urine sediment for differentiating glomerular and nonglomerular bleeding. Am J Kidney Dis 2008;52:235–41.

37. Eshbach ML, Kaur A, Rbaibi Y, Tejero J, Weisz OA. Hemoglobin inhibits albumin uptake by proximal tubule cells: implications for sickle cell disease. Am J Physiol Cell Physiol 2017;312:C733–40.

38. Gonzalez-Michaca L, Farrugia G, Croatt AJ, Alam J, Nath KA. Heme: a determinant of life and death in renal tubular epithelial cells. Am J Physiol Renal Physiol 2004;286:F370 –F377.

39. Schwartz GJ, Munoz A, Scheider MF, Mak RH, Kaskel F, Warady BA, Furth SL. New Equations to Estimate GFR in Children with CKD. J Am Soc Nephrol 2009;20:629–37.

40. Levey AS, Stevens LA, Schmid CH, Zhang YL, Castro AF, Feldman HI, et al. A new equation to estimate glomerular filtration rate. Ann Intern Med 2009;150:604–12.

Click here to access/download
Supporting Information
renamed_e56d3.pdf

Click here to access/download
Supporting Information
Consent.ehmn.docx

Click here to access/download
Supporting Information
Formulaire de consentement.ehmn.docx

Click here to access/download
Supporting Information
Base AA non DT versus SS globale.01.02.2020.ehmn.ac.xlsx

Click here to access/download
Supporting Information
Base AS.ehmn.plosone..xlsx
