# Supplementary material for: Biomarkers of sickle cell nephropathy in Senegal
Source: PLoS One. 2022 Nov 21;17(11):e0273745. doi: 10.1371/journal.pone.0273745 (PMC9678278; doi:10.1371/journal.pone.0273745)
Supplement: Attachment — Submitted filename: Review assignment for PONE-D-21-14690.docx [file pone.0273745.s007.docx]

**Review assignment for PONE-D-21-14690**

**BIOMARKERS OF SICKLE CELL NEPHROPATHY IN SENEGAL**

**PLOS ONE**

Sickle cell nephropathy is a common complication of SCD, which is one of the most frequent monogenic disorders worldwide. As Senegal is a country with high prevalence of the disease, studying the biomarkers of sickle cell nephropathy in Senegal is a relevant topic. However, there are major concerns regarding the methodology. Additionally, the results could have been reported more efficiently and in a less ambiguous way.

**Major concerns**

1 – Regarding the statistical methods, the authors do not clearly explain how the controls were selected. Although restriction to an ethno-linguistic group may be relevant, there is a lack of information about how the matching was performed.

2 – The definition presented for sickle cell disease is actually the definition for sickle cell anemia (patients whose phenotype is usually the most severe because of being homozygous for the beta S mutation).

3 – Please consider merging table 1 and 2 into a single table with columns for SCA patients, controls, and p-values. Keeping the number of observations for each variable is appropriate. However, median and interquartile range would be preferable to median (min-max) and 5-95^th^ percentiles).

**Minor concerns**

1 – The same keyword should be used throughout the paper when referring to “kidney dysfunction” for consistency. Avoid using dysfunction, impairment, abnormalities, etc., to refer to the same concept.

2 – The authors could consider using “without diabetes” instead of “free of diabetes”.

3 – The non-normally distributed continuous variables usually are presented as median (interquartile range). The interquartile range is the range from the 25^th^ to 75^th^ percentile, not the range from the 5^th^ to 95^th^ percentile which is presented by the authors.

4 – The authors should uniformize the way p-values are presented. For example, “p-value <0.001” instead of “p-value <10^-3” consistently.

5 – The sex distribution of the study sample should be presented as absolute and relative frequencies of the most common category/sex, instead of the female-to-male ratios.

6 – In line with the KDIGO guidelines, the albuminuria stages should be used instead of the older terms “microalbuminuria” and “macroalbuminuria”. Also, the term “chronic kidney disease” should be used instead “chronic renal insufficiency”.

7 - Glycosuria is a known marker of tubular dysfunction and the authors appropriately stress the importance of monitoring glycosuria as an early biomarker of sickle cell nephropathy. However, the term “microglucosuria” sounds inappropriate as it focus on the capacity of the laboratorial test to detect glycose in the urine.

8 – A high urine protein-to-creatinine ratio (UPCR) with normal urine albumin-to-creatinine ratio (UACR) is common in sickle cell nephropathy as a mechanism of tubular proteinuria is expected. The authors define tubular proteinuria as a UPCR-UACR ratio > 59%. The reason to use this cut-off should have been discussed. Please provide a reference for this.

9 – In table 4 avoid showing the observations for all categories of each variable as it is redundant. Please show the observations for the category of interest of each variable (for example, show the frequency of urinary albumin to creatinine ratio >= 30 mg/g). Also, the GFR categories could be presented in a decrescent order (e.g., > 140; 90-140; 60-90; <60).
