# Supplementary material for: Biomarkers of sickle cell nephropathy in Senegal
Source: PLoS One. 2022 Nov 21;17(11):e0273745. doi: 10.1371/journal.pone.0273745 (PMC9678278; doi:10.1371/journal.pone.0273745)
Supplement: Attachment — Submitted filename: Responses to ReviewersPONE-D-21-14690..docx [file pone.0273745.s008.docx]

**Response to Reviewers' comments**

We thank the editor and reviewers for their helpful comments. The manuscript has been revised to address their concerns, with additions and changes highlighted in blue. Please find their comments and our responses below each comment and bounded by dashed lines.

**Reviewers' Comments to Author:**

**Reviewer #1:**

Comments to the Author:

This is a well designed studies answering relevant questions for the chosen study population with potentially wider implications for the management of patients with SCD worldwide. The statistics, presentation of results and discussion are sound and logical.

---------------------------------------------------------------

Response: We thank the reviewer for this observation and his appreciation.

---------------------------------------------------------------

Point 1: I have made minor comments on the attached pdf.

---------------------------------------------------------------

Response 1: Thank you for these minor comments, these have been considered in the revised version of the paper which has now been edited, revising some English words and phrases.

---------------------------------------------------------------

Point 2: The main recommendation is for the authors to discuss a bit more the age difference between the cases and controls, whether that might have impacted findings and if so how

---------------------------------------------------------------

Response 2: We thank the reviewer for this recommendation.

Explanations on age and sex differences between cases and controls are now provided in the revised version in the discussion section of the manuscript. Moerever, in the statistical subsection in the Materials and Methods section, we have added a description of the matching process.

**Discussion**

Controls were recruited at random without any concern for matching on age and sex with cases. This recruitment method could explain the age and sex difference observed between the two groups and could also have had an impact on the comparative analysis results of biological indices “Table 3, Table 4”. To verify that the statistically significant differences noted with the first comparison did not result solely from a possible bias introduced by case-control age and sex difference, we carried out a second comparison, this time involving a subgroup of cases matched on age and sex to a subgroup of controls without any statistically significant difference regarding body mass index. This second comparison confirmed the results obtained with the age and sex unmatched groups “Table 3, Table 5”.

**Materials and methods**

Regarding matching, we proceeded in two steps. In the first step, we compared cases and controls without considering age and sex as matching parameters. In second step, we selected a subgroup of cases who matched on age and sex with a subgroup of controls without any statistically significant difference regarding body mass index. Then these two subgroups were compared. In both steps, cases and controls was ethnic matched: all subjects included in both groups were sub-Saharan African black people.

---------------------------------------------------------------

**Reviewer #2:**

Comments to the Author:

Sickle cell nephropathy is a common complication of SCD, which is one of the most frequent monogenic disorders worldwide. As Senegal is a country with high prevalence of the disease, studying the biomarkers of sickle cell nephropathy in Senegal is a relevant topic. However, there are major concerns regarding the methodology. Additionally, the results could have been reported more efficiently and in a less ambiguous way.

---------------------------------------------------------------

Response: We thank the reviewer for his concerns. These have been addressed point by point below and the paper has now been revised to consider different concerns raised.

---------------------------------------------------------------

**Major concerns**

1 – Regarding the statistical methods, the authors do not clearly explain how the controls were selected. Although restriction to an ethno-linguistic group may be relevant, there is a lack of information about how the matching was performed.

---------------------------------------------------------------

Response 1: Thank you to the reviewer for these observations.

Explanations on age and sex differences between cases and controls are now provided in the revised version in the discussion section of the manuscript. Moerever, in the statistical subsection in the Materials and methods section, we have added a description of the matching process.

**Discussion**

Controls were recruited at random without any concern for matching on age and sex with cases. This recruitment method could explain the age and sex difference observed between the two groups and could also have had an impact on the comparative analysis results of biological indices “Table 3, Table 4”. To verify that the statistically significant differences noted with the first comparison did not result solely from a possible bias introduced by case-control age and sex difference, we carried out a second comparison, this time involving a subgroup of cases matched on age and sex to a subgroup of controls without any statistically significant difference regarding body mass index. This second comparison confirmed the results obtained with the age and sex unmatched groups “Table 3, Table 5”.

**Materials and methods**

Regarding matching, we proceeded in two steps. In the first step, we compared cases and controls without considering age and sex as matching parameters. In second step, we selected a subgroup of cases who matched on age and sex with a subgroup of controls without any statistically significant difference regarding body mass index. Then these two subgroups were compared. In both steps, cases and controls was ethnic matched: all subjects included in both groups were sub-Saharan African black people.

---------------------------------------------------------------

2 – The definition presented for sickle cell disease is actually the definition for sickle cell anemia (patients whose phenotype is usually the most severe because of being homozygous for the beta S mutation).

---------------------------------------------------------------

Response 2: We thank the reviewer for this comments. The definition have been adjusted in the revised version.

Sickle cell disease (SCD) refers to a collection of inherited blood disorders that feature the propensity for erythrocytes to change into crescent or so-called sickle shapes (Ware, 2017).

Ware RE, de Montalembert M, Tshilolo L, Abboud MR. Sickle Cell Disease. Lancet 2017 ; 390 (10091): 311-323.

---------------------------------------------------------------

3 – Please consider merging table 1 and 2 into a single table with columns for SCA patients, controls, and p-values. Keeping the number of observations for each variable is appropriate. However, median and interquartile range would be preferable to median (min-max) and 5-95^th^ percentiles).

---------------------------------------------------------------

Response 3: We thank the reviewer for different observations

We have taken note of your comment. However, we would like to explain the reasons why we have presented these two tables separately.

The results presented in Table 1 and 2 summarise anthropometric and biological characteristics of the two groups. In particular, Table 1 shows 5^th^ and 95^th^ percentiles of controls that are essential to identify values that could be considered as pathological in the absence of a cut-off of a consensus as it exists for albuminuria or glomerular filtration rate (Nader Rifai, 2018). Regarding your concern, we believe that it has been taken into account in Tables 3, 4 and 5 which present the results of the comparative analysis between the two groups (cases and controls) while indicating the p-values.

As you mentioned in your comments, we can summarise a distribution whose normality is in doubt using parameters such as median, extremes (minimum and maximum) and quantiles (quartiles, deciles, centiles especially percentiles). In our study, we chose to present the 5^th^ and 95^th^ percentiles as the information we could obtain from them would contribute to the achievement of one of our objectives which was to establish cut-off of certain biological indices, in particular that of glucosuria (table 1) (Nader Rifai et al., 2018).

With regard to the biological indices presented in tables 4 and 5, we have chosen not to compare their averages as we did for glycemia or creatininemia because if this were the case the information derived would be, for instance, the mean albuminuria of sickle cell patients was increased compared to that of controls. This is not necessarily clinically relevant for us. For this reason, we preferred to transform them into clinically relevant categorical variables. Thus, we could not only know that albuminuria is increased in sickle cell disease but also that this increase is due to the fact that microalbuminuria is more frequent in sickle cell patients compared to controls and normoalbuminuria is more prevalent in controls compared to sickle cell patients.

[Nader Rifai, Andrea Rita Horvath, Carl T. Wittwer (2018). Tietz fundamentals of clinical chemistry and molecular diagnostics](https://www.google.com/search?sxsrf=ALeKk03Bb5_Ow0h5SY9TbgzVa4TAXNls0w:1629644506721&q=Derniere+edition+Tiez+text+book+fundamentals+of+clinical+chemistry+and+molecular+diagnostics&nfpr=1&sa=X&ved=2ahUKEwionbvv8sTyAhUbXc0KHTBmD4EQvgUoAXoECAIQNQ), 8^th^ edition, **p.375**, Elsevier. we have scanned and joined this page.

---------------------------------------------------------------

**Minor concerns**

1 – The same keyword should be used throughout the paper when referring to “kidney dysfunction” for consistency. Avoid using dysfunction, impairment, abnormalities, etc., to refer to the same concept.

---------------------------------------------------------------

Response 1: We thank the reviewer for this observation. These different annotations have now been harmonized.

---------------------------------------------------------------

2 – The authors could consider using “without diabetes” instead of “free of diabetes”.

---------------------------------------------------------------

Response 2: This has been adjusted where applicable.

---------------------------------------------------------------

3 – The non-normally distributed continuous variables usually are presented as median (interquartile range). The interquartile range is the range from the 25^th^ to 75^th^ percentile, not the range from the 5^th^ to 95^th^ percentile which is presented by the authors.

---------------------------------------------------------------

Response 3: As we had to explain in response to question 3 raised in the major concerns, our choice to use percentiles was guided essentially by the achievement of one of our objectives, namely to establish cut-off of certain biological indices, in particular that of glucosuria.

---------------------------------------------------------------

4 – The authors should uniformize the way p-values are presented. For example, “p-value <0.001” instead of “p-value <10^-3” consistently.

---------------------------------------------------------------

Response 4 : Thank you to the reviewer for pointing this out. Different p-values have now been made uniform, opting for decimal representation, as suggested.

---------------------------------------------------------------

5 – The sex distribution of the study sample should be presented as absolute and relative frequencies of the most common category/sex, instead of the female-to-male ratios.

---------------------------------------------------------------

Response 5: This observation has been considered in the revised version of the manuscript.

---------------------------------------------------------------

6 – In line with the KDIGO guidelines, the albuminuria stages should be used instead of the older terms “microalbuminuria” and “macroalbuminuria”. Also, the term “chronic kidney disease” should be used instead “chronic renal insufficiency”.

---------------------------------------------------------------

Response 6: Thank you to the reviewer for different observations.

As regards chronic renal insufficiency, we have replaced it in the text by chronic kidney disease.

You are quite right to recommend that we use the term albuminuria stage A2 to be in line with the current guidelines issued since 2012 by the KDIGO. We hesitated to do so for two reasons: the first is that the term microalbuminuria is still widely used by many authors of recent publications on sickle cell nephropathy in respected journals (e.g. : Ranque B, 2014; Geard A, 2017; Niss O, 2020); and the second and main reason is that we aimed to define microglucosuria based on the definition of microalbuminuria. So we felt it was important to keep the term microalbuminuria. But as you have pointed out we propose to keep the term albuminuria stage A2 by putting in brackets (microalbuminuria) where it seems necessary for us.

Ranque B, Menet A, Diop IB, Thiam MM, Diallo D, Diop S, et al. [Early renal damage in patients with sickle cell disease in sub-Saharan Africa: a multinational, prospective, cross-sectional study](https://www.sciencedirect.com/science/article/pii/S2352302614000076). Lancet Haematol 2014;1:e64–73.

[Geard](https://pubmed.ncbi.nlm.nih.gov/?term=Geard+A&cauthor_id=28466968) A, [Pule](https://pubmed.ncbi.nlm.nih.gov/?term=Pule+GD&cauthor_id=28466968) GD, [Chemegni](https://pubmed.ncbi.nlm.nih.gov/?term=Chetcha+Chemegni+B&cauthor_id=28466968) BC, [Bitoungui](https://pubmed.ncbi.nlm.nih.gov/?term=Ngo+Bitoungui+VJ&cauthor_id=28466968) VJN, [Kengne](https://pubmed.ncbi.nlm.nih.gov/?term=Kengne+AP&cauthor_id=28466968) AP, [Chimusa](https://pubmed.ncbi.nlm.nih.gov/?term=Chimusa+ER&cauthor_id=28466968) ER, [Wonkam](https://pubmed.ncbi.nlm.nih.gov/?term=Wonkam+A&cauthor_id=28466968) A. Clinical and genetic predictors of renal dysfunctions in sickle cell anaemia in Cameroon. Br J Haematol 2017;178:629-39.

Niss O, Lane A, Asnani MR, Yee ME, Raj A, Creary S et al. Progression of albuminuria in patients with sickle cell anemia: a multicenter, longitudinal study. Blood Advances 2020; 4: 1501-1511.

---------------------------------------------------------------

7 - Glycosuria is a known marker of tubular dysfunction and the authors appropriately stress the importance of monitoring glycosuria as an early biomarker of sickle cell nephropathy. However, the term “microglucosuria” sounds inappropriate as it focus on the capacity of the laboratorial test to detect glycose in the urine.

---------------------------------------------------------------

Response 7:

In the latest edition of a reference book in clinical chemistry (Nader Rifai et al, 2018), we can read on page 362 the following: “Microalbuminuria is a term that has been widely used to described an increase in urinary albumin loss above the reference interval for healthy non diabetic subjects, but at a level that is not generally detectable by less sensitive clinical tests such as reagent strips designed to measure total protein. It is also in similar terms that Viberti et al., in their article that has become a reference « microalbuminuria as a predictor of clinical nephropathy in insulin-dependent diabetes mellitus » published in Lancet in 1982 defined microalbuminuria: Microalbuminuria is defined as the presence of small amounts of albumin in the urine, less than 500 mg/24 h (300 mg/L) proteinuria (therefore undetectable by traditional proteinuria screening methods including test strips) but greater than physiological proteinuria (30 mg/24 h).

We have used this definition of microalbuminuria to define microglucosuria by analogy. Indeed, in the article we defined microglucosuria as follows: Microglucosuria was defined as glucosuria which is not a consequence of hyperglycemia and which might not be detectable by urine test strips that generally do not detect glucosuria below 50 mg/dl (2.775 mmol/l) but which is quantifiable by the glucose oxidase peroxidase method which can determine glucosuria 200 times lower (0.23 mg/dl [0.013 mmol/l]) according to the manufacturers of the reagents used in our study. Glucosuria greater than or equal to the 95^th^ percentile of the UGCR in the control group was considered to be microglucosuria. And, according to our results the 95th percentile in the control subjects was 20 mg/g creatinine.

So, we propose to keep the term microglucosuria in the text.

[Nader Rifai, Andrea Rita Horvath, Carl T. Wittwer (2018). Tietz fundamentals of clinical chemistry and molecular diagnostics](https://www.google.com/search?sxsrf=ALeKk03Bb5_Ow0h5SY9TbgzVa4TAXNls0w:1629644506721&q=Derniere+edition+Tiez+text+book+fundamentals+of+clinical+chemistry+and+molecular+diagnostics&nfpr=1&sa=X&ved=2ahUKEwionbvv8sTyAhUbXc0KHTBmD4EQvgUoAXoECAIQNQ), 8^th^ edition, **p.362,** Elsevier. *We have scanned and joined this page.*

[Viberti](https://pubmed.ncbi.nlm.nih.gov/?term=Viberti+GC&cauthor_id=6123720) GC,  [Hill](https://pubmed.ncbi.nlm.nih.gov/?term=Hill+RD&cauthor_id=6123720) RD,  [Jarrett](https://pubmed.ncbi.nlm.nih.gov/?term=Jarrett+RJ&cauthor_id=6123720) RJ,  [Argyropoulos](https://pubmed.ncbi.nlm.nih.gov/?term=Argyropoulos+A&cauthor_id=6123720) A,  [Mahmud](https://pubmed.ncbi.nlm.nih.gov/?term=Mahmud+U&cauthor_id=6123720) U, [Keen](https://pubmed.ncbi.nlm.nih.gov/?term=Keen+H&cauthor_id=6123720) H. Microalbuminuria as a predictor of clinical nephropathy in insulin-dependent diabetes mellitus Lancet 1982, 1(8287):1430-2.

---------------------------------------------------------------

8 – A high urine protein-to-creatinine ratio (UPCR) with normal urine albumin-to-creatinine ratio (UACR) is common in sickle cell nephropathy as a mechanism of tubular proteinuria is expected. The authors define tubular proteinuria as a UPCR-UACR ratio > 59%. The reason to use this cut-off should have been discussed. Please provide a reference for this.

---------------------------------------------------------------

Response 8: Thank you to the reviewer for this comment.

Articles 34 and 38 are provided in the text as a reference for the use of this cut-off.

34. Bökenkamp A. Proteinuria—take a closer look! Pediatr Nephrol 2020;35:533–41.

38. Ohisa N, Yoshida K, Matsuki R, Suzuki H, Miura H, Ohisa Y, et al. A comparison of urinary albumin-total protein ratio to phase-contrast microscopic examination of urine sediment for differentiating glomerular and nonglomerular bleeding. Am J Kidney Dis 2008;52:235–41.

---------------------------------------------------------------

9 – In table 4 avoid showing the observations for all categories of each variable as it is redundant. Please show the observations for the category of interest of each variable (for example, show the frequency of urinary albumin to creatinine ratio >= 30 mg/g). Also, the GFR categories could be presented in a decrescent order (e.g., > 140; 90-140; 60-90; <60).

---------------------------------------------------------------

Response 9: We thank the reviewer for these comments. Observations are shown only for the category of interest and the GFR categories are now presented in a descending order, as suggested.

---------------------------------------------------------------
