# Supplementary material for: Biomarkers of sickle cell nephropathy in Senegal
Source: PLoS One. 2022 Nov 21;17(11):e0273745. doi: 10.1371/journal.pone.0273745 (PMC9678278; doi:10.1371/journal.pone.0273745)
Supplement: Attachment — Submitted filename: REBUTTAL LETTER.docx [file pone.0273745.s010.docx]

**REBUTTAL LETTER**

**Date**: July 19, 2022

**Place**: Dakar, Senegal

**From**

Dr El Hadji Malick NDOUR

Department of Pharmaceutical Biochemistry

Faculty of Medicine, Pharmacy and Dentistry

Cheikh Anta Diop University

Albert Royer National Hospital of Children

B.P. 25755 Dakar-Fann

Tel./Mobile Number: +221 70 206 17 27

[elhadjimalickndour@yahoo.fr](mailto:elhadjimalickndour@yahoo.fr)

[elhadjimalick1.ndour@ucad.edu.sn](mailto:elhadjimalick1.ndour@ucad.edu.sn)

**To**

**The Editor-in-Chef**

*Plos One*

**Sub: Revised Manuscript PONE-D-21-14690R4 Submission**

**Article Type**: Research Article

**Dear Sir**,

Greetings

We thank you for reviewing our revised manuscript PONE-D-21-14690R3 and for your appreciation.

Please find attached soft copy of our manuscript PONE-D-21-14690R4 entitled “BIOMARKERS OF SICKLE CELL NEPHROPATHY” for publication in your esteemed journal *Plos One*.

Please find below our responses to the editor and reviewer’s comments.

-----------------------------------

**Response to Reviewers' comments**

We thank the editor and reviewers for their helpful comments. The manuscript has been revised to address their concerns, with additions and changes highlighted in yellow. Please find their comments and our responses below each comment and bounded by dashed lines.

**Reviewers' Comments to Author:**

**Reviewer #4:**

**Comments to the Author:**

 El Hadji Malick Ndour et al performed case-control study to identify the candidate biomarkers for sickle cell nephropathy in sickle cell anemia from Senegal population. A total of 163 patients with SCA and 177 ethnic matched controls were enrolled. The higher prevalence of hyposthenuria, glomerular hyperfiltration, microalbuminuria, proteinuria, tubular proteinuria, and microglucoruia were observed in SCA patients compared with controls. Although the study is rather descriptive, the study to investigate the prevalence of renal dysfunction in SCA patients is unique and interesting. However, there are several concerns as follow.

-----------------------------------------------------------------------------------------------------

**Response:** We thank the reviewer for his appreciation and observation.

**Major comments**

**Point 1**

In abstract line 5, what is the definition and diagnostic criteria for “sickle cell nephropathy”? The authors should apply the diagnostic criteria for “sickle cell nephropathy” in the SCA patients. In addition, how did the authors exclude other kidney diseases in SCA and control groups?

**Response 1:** We thank the reviewer for his questions and his recommendations.

The definition and diagnostic criteria for sickle cell disease involve radiological and histological parameters in addition to biological parameters (albuminuria and glomerular filtration rate). Radiological and histological parameters were not available for the study. Therefore, we tried instead to identify biological indices abnormalities that can help to indicate kidney dysfunction.

In the discussion section, we pointed out that among the limitations of this study we did not look for infectious etiologies of nephropathy : “The serologic assays of the *hepatitis B virus*, *Streptococcus pneumoniae or Schistosoma haematobium* had have not been carried out though these pathogens may cause the kidney dysfunction in some controls or patients with SCA”.

-----------------------------------------------------------------------------

**Point 2**

In line 63-65 Table 1 and 2, were the patients with diabetes excluded? Were there no patients with blood glucose levels more than 170-180 mg/dL?

---------------------------------------------------------------------------

**Response 2:** We thank the reviewer for this question.

None of the sickle cell patients or controls included in this study had a blood glucose level more than 126 mg/dl (which is the threehold to define diabetes), hence 170-180 mg/dl as we indicated in the materials and methods section

“Patients with SCA were included in the study if they were already enrolled in the sickle cell adult or children cohort, at least 4 years of age, at a routine fasting visit, and in steady state health. The exclusion criteria included those in a pain crisis and/or with diabetes. Samples from control participants were collected when they were apparently healthy and at least 4 years old. Control participants were excluded from the study when their hemoglobin solubility test was positive and their *HBB* genotype was β^S^/β^A^ and/or their fasting blood sugar ≥ 1.26 g/l”.

-----------------------------------------------------------------

**Point 3**

In lines 91 and 272-274, the authors should describe the definition of glomerular hyperfiltration.

-------------------------------------------------------------------------------------

**Response 3:** We thank the reviewer for this recommandation.

We have described the definition of glomerular hyperfiltration in materials and methods section :

In line 401, “Glomerular hyperfiltration (GHF) was defined by GFR > 140 ml/min/1.73m²”.

---------------------------------------------------------------------------

**Point 4**

In line 92, the authors should describe the definition of chronic kidney disease (CKD).

-------------------------------------------------------------------------------------------------

**Response 4** : We thank the reviewer for this recommandation.

Chronic kidney disease (CKD) have been replaced with renal failure and its definition have been given in materials and methods section :

In line 401-402, “...and renal failure by GFR < 60 ml/min/1.73m²”.

--------------------------------------------------------------------------------------

**Point 5**

 In line 93, did the authors measure tubular injury markers such as β2-microglobulin?

-----------------------------------------------------------------------------------------------------

**Response 5 :** We thank the reviewer for this question.

No, we did not measure β2-microglobulin. Biomarkers of renal tubular lesions (β2-microglobulin, α1-microglobulin, N-acetylglucosaminidase, retinol binding protein (RBP)) are not yet available in Senegal.

---------------------------------------------------------------------------------------

**Point 6**

In lines 306-309, how did the authors statistically match the age and sex? Did the authors use the propensity score matching?

-------------------------------------------------------------------------------------------------------------

**Response 6:** We thank the reviewer for this questions

-In line 412-417, we have described how we proceeded for matching.

“Regarding matching, we proceeded in two steps. In the first step, we compared cases and controls without considering age and sex as matching parameters. In second step, we selected a subgroup of cases who matched on age and sex with a subgroup of controls without any statistical significant difference regarding body mass index. Then these two subgroups were compared. In both steps, cases and controls was ethnic matched: all subjects included in both groups were sub-Saharan African black people”.

-We did not use the propensity score matching.

--------------------------------------------------------------------------------------------

**Point 7**

If the authors are able to make the diagnosis “sickle cell nephropathy” according to the diagnostic criteria, the authors investigate the sensitivity, specificity and AUC of cut-off points of hyposthenuria, glomerular hyperfiltration, microalbuminuria, proteinuria, tubular proteinuria, and microglucoruia for the diagnosis of “sickle cell nephropathy”.

---------------------------------------------------------------------------------------------------------------

**Response 7**: We thank the reviewer for this recommandation.

As we have explained in Response 1, we were not able to make the diagnosis because of lake of information regarding radiological and and histological parameters.

**Reviewer #5:**

**Comments to the Author:**

The authors showed a relatively high proportion of SCA nephropathy among patients living with SCA in Senegal, and several parameters were suggested as prevalent biomarkers of nephropathy. This article seems to be refined after appropriate revision, and the contents is interesting. However, I regret to realize several problems of the article.

-------------------------------------------------------------------------------------------------

**Response:** We thank the reviewer for his appreciation and concerns. These have been addressed point by point below to consider different concerns raised.

----------------------------------------------------------------------------

**Point 1**

This is a critical problem. The authors ignored the effect of the skeletal muscle volume on serum creatinine levels and urinary creatinine excretion. The skeletal muscle volume was known to decrease in patients with SCA in both child and adult (Barden et al. Am J Clin Nutr. 2002; 76: 218-25., Ravelojaona et al. Am J Pathol. 2015; 185: 1448-56.). Both serum creatinine levels and urinary creatinine excretion decrease in patient with lower skeletal muscle volume. Hence, eGFR, UACR and UPCR would be overestimated in patients with SCA. Actually, although statistical difference for BMI was not observed among age- and sex-matched patients, it strongly tended to lower in patients with SCA (Table 3: p=0.079). BMI would decrease in patients with lower skeletal muscle volume. Because patient number was relatively small, hence beta error for BMI should be considered.

------------------------------------------------------------------------------------------------------------

**Response 1:** We thank the reviewer for this observation.

We fully agree with this observation. However, despite all the concerns raised, creatinine continues to be used to assess the kidney function of sickle cell patients in major sickle cell disease centres ([15], [28], [37]...). This is because alternative parameters are not routinely available. An alternative parameter is cystatin C but it is not yet available in Senegal and its cost is not affordable, especially in our context of a country with limited resources. In our study, we did not use serum creatinine alone or creatinine clearance to assess changes in glomerular filtration rate even if it was possible to do so. To avoid concerns raised, we used formulae instead. These formulae were developed taking into account parameters that may influence creatinine production [59, 60]. These equations have been shown to be effective when compared to reference methods in sickle cell subjects ([34], [35]...).

Furthermore, the use of creatinine to normalise urine concentrations of proteins, albumin and glucose remains problematic. However, to date, it is the only parameter accepted to normalize the concentration of these parameters in urine when a 24 hours urine collection is not possible. In any case, the collection of 24 hours urine has real limits because it depends on the goodwill of the patients. Moreover, in the context of sickle cell disease, with the enuresis that accompanies the loss of the kidneys' concentrating ability, we can never be sure that the volume collected is complete. In addition, asking patients to collect 24 hours urine would also mean asking patients to return to hospital with their own means, since in this study we did not have funds to pay for the transport of patients.

----------------------------------------------------------------------------

**Point 2**

This is a second critical problem. The timing to collect urine for urinary analysis should be described. Because amount of water drinking or food intake can affect USG, especially in non-early morning urine.

----------------------------------------------------------------------------------------------

**Response 2:** Thank you to the reviewer for these observations.

Venous blood and random midstream urine specimens were collected after at least 8 hours of fasting. (This duration of fasting was also necessary to measure fasting plasma glucose in order to exlude patient with diabetes).

This has been added in the manuscript in materials and methods section.

------------------------------------------------------------------------------------------

**Point 3**

Did the authors evaluate urinary volume, amount of drinking and serum sodium concentration? These parameters would be different in patients with SCA because impairment of urinary concentration was observed.

--------------------------------------------------------------------------------------------------------------

**Response 3:** Thank you to the reviewer for this question and comment.

No, we did not. We share your opinion.

-------------------------------------------------------------------------------------------------------------

**Point 4**

Which one was the most important biomarker for detect SCA nephropathy? Was there any difference between early and late phase? Which biomarker was associated with poor renal prognosis?

-----------------------------------------------------------------------------------------------------------

**Response 4:** Thank you to the reviewer for these questions.

In this study, we limited to looking for biomarkers indicating alteration of a specific renal functions and renal structural lesions. GFR and microalbuminuria are two biomarkers that are widely used in the contexte of SCA to evaluate kidney functions.

We did not investigate early or late stages and prognosis of sickle cell nephropathy.

----------------------------------------------------------------------------------------------------

**Point 5**

I may be wrong but could you confirm line 172 and 185? Was it "Table 5" instead of "Table 4"? Because the authors described “Table 5” in both sentences, but the values for the prevalence of GHF and CKD, which were described in line 171 and 183, were same to the values described in Table 4.

**Respone 5:** Thank you to the reviewer for different observations. References have been now adjusted.

----------------------------------------------------------------------------------------------------------

**Point 6**

I may be wrong but could you confirm the value for blood pressure? I think “regular” blood pressure is about 120/70 mmHg, but the described value seems one digit missing.

-----------------------------------------------------------------------------------------------------------

**Response 6:** Thank you to the reviewer for pointing this out. Values of blood pressures have been adjusted.

----------------------------------------------------------------------------------------------------------

**Reviewer #6:**

Dear authors,

Thank you for this submission.

My comment are given below.

------------------------------------------------------------------------------------------------------------

**Reponse:** We thank the reviewer for his comments. These have been addressed point by point below and the paper has now been revised to consider different concerns raised.

-------------------------------------------------------------------------------------------

**Abstract**
The terms creatininemia nad creatinuria do not sound right as everyone has creatinine in blood and urine. They are not used widely. Please revise term with known, widely used and accepted ones. This is valid for these terms through all manuscript. My suggestion is creatinine and urine creatinine.

--------------------------------------------------------------------------------------------------------------

**Response :** We thank the reviewer for this observation.

The terms creatininemia and creatininuria have been replaced with serum creatinine and urine creatinine in the manuscript.

--------------------------------------------------------------------------------------------

**Introduction**
Details about sickle cell disease are very important but as the topic is sickle cell nephropathy they could be summarized to highlight nephropathy more.

----------------------------------------------------------------------------------------------------------------

**Response:** We thank the reviewer for this recommantions.

We think you are right. Nevertheless, we would like to inform about our unfavorable context of sickle cell disease management, in order to better justify the choice of some parameters that we have proposed to use as biomarkers to assess the renal function of sickle cell patients, rather than other parameters considered as reference ones.

---------------------------------------------------------------------------------------------

**Results**
I think there is no need to give 5-95 percentiles at table 1 and 2. Please revise them and unite table 1 and 2 which would make them easily seen together and comparable. In addition, a column could be added to this table for p values which would make table 3 unnecessary as well. There is no need to define controls as AA non-DT as details of them are given at the text. It may be refereed just as control group. In summary, in my opinion, a table for all participants’ values with p values and another table for just sex and age matched controls containing all parameters would be more suitable. P values should be present for all parameters given at tables, not for some parameters.

Blood pressures are written to be in mmHg but there should a mistake at numbers. They are so small and I think are given in cmHg. Please check them. Details between line 75-80 are methodological ones so they should be removed to methods section. There should be no reference given at results section. Please revise sentences between lines 91 and 103. Adding ‘table…’ to the end of long sentences are not grammatically correct. Parameters could be given one by one and detailed according to the groups, sickle cell and control, and p value could be given at last. Moreover, there should be consistency between definition of parameters at the text and at the table. If you mention about albuminuria stage 2 at the text, a reader must see this parameter when looking to the table easily. Revise text and tablet o make them parallel to each other. Instead of stage 2 albuminuria, microalbuminuria could be used if you prefer.
When I read results section, it made my mind confused rather than being aware of differences between patients and controls. It should be more precise.

-----------------------------------------------------------------------------------------------------------------

**Response:** We thank the reviewer for different observations and suggestions.

-We have taken note of your comment. However, we would like to explain the reasons why we have presented these two tables separately. The results presented in Table 1 and 2 summarise anthropometric and biological characteristics of the two groups. The purpose of this two tables is not to compare the two groups. Table 1 shows 5^th^ and 95^th^ percentiles of controls that are essential to identify values that could be considered as pathological in the absence of a cut-off of a consensus as it exists for albuminuria or glomerular filtration rate (Nader Rifai et al., 2018). Thus, we were able to set cut-off for glucosuria and to define microglucosuria and hyposthenuria. The 5^th^ and 95^th^ percentiles of albuminuria, proteinuria and GFR testify to the reliability of the method as they gave values comparable to those already used to interpret routine patients test results (Nader Rifai et al., 2018).

[Nader Rifai, Andrea Rita Horvath, Carl T. Wittwer (2018). Tietz fundamentals of clinical chemistry and molecular diagnostics](https://www.google.com/search?sxsrf=ALeKk03Bb5_Ow0h5SY9TbgzVa4TAXNls0w:1629644506721&q=Derniere+edition+Tiez+text+book+fundamentals+of+clinical+chemistry+and+molecular+diagnostics&nfpr=1&sa=X&ved=2ahUKEwionbvv8sTyAhUbXc0KHTBmD4EQvgUoAXoECAIQNQ), 8^th^ edition, **p.375**, Elsevier. we have scanned and joined this page.

-We have replaced AA non-DT with controls, where appropriate.

-Regarding the missing p-values in Tables 4 and 5 (on the line of normal glomerular filtration), p-value was not given because this sub-group is considered to be the reference one; p-values were computed with respect to this group. Therefore, it does not have p-value.

-Values of blood pressures have been corrected.

-Details between line 75-80 have been removed to the methods section.

-We may be wrong but we cannot find any references that we would have indicated in the results section.

-Sentences between line 91 – 103 have been revised.

-Texts and tables have now been harmonised.

-We have tried to describe differences between SCA patients and controls more precisely.

----------------------------------------------------------------------------------------------------

**Discussion**
This section should not repeat findings. To refer a finding, a short sentence referring findings is enough and there should be some comment or speculation about it besides comparison with other similar studies. Please try to remove repetitions regarding finding as much as possible.
GFR was found by different formula in children and adults. Is it correct to put GFRs got by two different formula and present them together? I think it is not correct. The percentage of children is not given. You should give this detail. If you would like to present GFRs together, you should add this a limitation. Another limitation is the selection of control group. It is open to bias, especially sex and age matched one. It should be also added as a limitation. The details of GFR measurements should be transferred to methods section. At discussion, the reason for this and its possible effects.

-------------------------------------------------------------------------------------------------

**Response**

-Repetitions regarding finding have been removed, where appropriate, in the discussion section.

-We have added the percentage of children in the results section.

-Limitations regarding the GFR and the selection of the control group have been taken into account in the text

-The details of the GFR measurements have been moved to the methods section and the reason for using different formulae has been added to the discussion section.
